# Recent Advances in the Development of Drug Delivery Applications of Magnetic Nanomaterials

**DOI:** 10.3390/pharmaceutics15071872

**Published:** 2023-07-03

**Authors:** Alexandra Pusta, Mihaela Tertis, Izabell Crăciunescu, Rodica Turcu, Simona Mirel, Cecilia Cristea

**Affiliations:** 1 Department of Analytical Chemistry and Instrumental Analysis, Iuliu Hațieganu University of Medicine and Pharmacy, 4 Louis Pasteur Street, 400349 Cluj-Napoca, Romania; alexandra.pusta@umfcluj.ro (A.P.); mihaela.tertis@umfcluj.ro (M.T.); 2 Department of Medical Devices, Iuliu Hațieganu University of Medicine and Pharmacy, 4 Pasteur Street, 400349 Cluj-Napoca, Romania; smirel@umfcluj.ro; 3 National Institute for Research and Development of Isotopic and Molecular Technologies, 400293 Cluj-Napoca, Romania; izabell.craciunescu@itim-cj.ro (I.C.); rodica.turcu@itim-cj.ro (R.T.)

**Keywords:** magnetic nanomaterials, cancer, drug delivery systems, targeted therapy, personalized treatment

## Abstract

With the predicted rise in the incidence of cancer, there is an ever-growing need for new cancer treatment strategies. Recently, magnetic nanoparticles have stood out as promising nanostructures for imaging and drug delivery systems as they possess unique properties. Moreover, magnetic nanomaterials functionalized with other compounds can lead to multicomponent nanoparticles with innovative structures and synergetic performance. The incorporation of chemotherapeutic drugs or RNA in magnetic drug delivery systems represents a promising alternative that can increase efficiency and reduce the side effects of anticancer therapy. This review presents a critical overview of the recent literature concerning the advancements in the field of magnetic nanoparticles used in drug delivery, with a focus on their classification, characteristics, synthesis and functionalization methods, limitations, and examples of magnetic drug delivery systems incorporating chemotherapeutics or RNA.

## 1. Introduction

Cancer represents a major public health problem worldwide, being the first leading cause of death in people below the age of 70 in North America, Canada, Australia, China, and numerous European countries [1]. With the increase in population and life expectancy, it is predicted that the incidence of cancer will rise in the following years, reaching 28.4 million cases annually worldwide by 2040 [2]. In this context, finding effective therapies for cancer is crucial for reducing the medical and economic burden of this disease.

Conventional cancer therapies include surgery, radiotherapy, and/or chemotherapy. Among these, chemotherapy is widely used but presents various side effects that can limit patient compliance and adherence [3]. Moreover, cancer cells can develop chemotherapy resistance through many mechanisms, rendering it ineffective [4]. Recently, non-coding RNAs such as micro RNAs (miRNA) and small interfering RNAs (siRNA) have been studied for their potential as novel cancer treatments due to their capacity to regulate gene transcription [5]. However, their applicability is limited too by their low stability, high costs, and immunological adverse reactions [6].

In recent years, nanotechnology has emerged as a tool for cancer treatment, promising improved therapeutic outcomes for both chemotherapy and RNA-based therapy, by delivering therapeutic agents in close proximity to the tumor using nano drug delivery systems (DDSs). Nano DDSs generally range in size between 10 and 100 nm, which allows sufficient circulation time and accumulation in the tumor tissue due to the enhanced permeability and retention (EPR) effect [7,8]. Numerous types of DDSs have been employed, such as polymers [9,10], lipid nanoparticles (NPs) [11], and metallic NPs [12] (silver, gold, or magnetic NPs). Among these, magnetic NPs represent a promising alternative, due to their properties such as high stability, high saturation magnetization/large magnetic moment of particles, good response to moderate magnetic fields, inherent ability to cross biological barriers, protection of the drug from rapid degradation in biological systems, provision of a large surface area for conjugating targeting ligands [7,8,13,14,15,16,17,18,19], low production costs [20], and superparamagnetism, which allows their guidance in the organism using an external magnetic field [7,21]. Scientific interest in NPs in general and in magnetic nanoparticles (MNPs) in particular has grown exponentially in the last decade, due to recent high-interest research on their properties and the fact that in a relatively short time, these materials have become particularly important tools in high-interest biomedical areas such as biomaterial science, biochemistry, diagnostics, magnetic drug and gene delivery, hyperthermia, magnetic resonance imaging (MRI), and theragnostics [22,23,24,25,26,27,28,29,30,31]. The increased interest in the field of MNPs is demonstrated by the number of scientific articles provided by a simple “magnetic nanoparticles” keyword search in the Scopus database. Before 1995, fewer than 100 articles were published, but after 1996, when the first successful clinical trials took place, there was an exponential increase with 3000 articles in 2010, 6500 in 2015, and up to 8700 in 2020 [32]. As a general structure, NPs are considered inorganic or organic particles of submicron size with enhanced properties relative to a similar bulk form. The specific physical and chemical properties given by nanostructuring, such as optical, electrical, and magnetic properties or increased reactivity, made these special types of NPs attractive to nanotechnology. Following the development of biotechnological applications, the term “bio-magnetic nanoparticles” (BMNPs) was introduced, describing a unique combination of physio-chemical properties of magnetic nanoparticles with their entirely biocompatible nature, which makes them particularly effective in various biomedical applications [33]. Starting from the particular requirements of each biomedical application, BMNPs are considered to have a huge potential in drug delivery applications, because their surface can be specifically functionalized with various molecular layers [7,14,24,34]. Apart from drug delivery, magnetic NPs can be used for hyperthermia applications, MRI contrast imaging, and diagnosis procedures. Recently, magneto-mechanical actuation of MNPs has also been used as an anti-cancer strategy. In this technique, the application of a magnetic field does not lead to heating (such as in hyperthermia), but to vibrations of the MNPs in the proximity of cells, leading to mechanical alterations and cell death [35,36]. Moreover, complex strategies combining more of these approaches can also be employed. The combination of hyperthermia and drug delivery in the same carrier can enhance anti-cancer efficiency destroying cancer cells through multiple mechanisms. Theragnostic approaches also represent a promising direction, combining simultaneous treatment and diagnosis for improved cancer management. These applications have been extensively covered elsewhere [7,13,20,37,38] and do not represent the scope of this review.

In this work, we will present the main types of magnetic nanomaterials and their characteristics, classification, and common synthesis procedures. A few limitations of MNPs and some considerations on the development of DDSs will be presented. In the next section, we will present and comparatively discuss examples of applications of magnetic nanomaterials used for the targeted delivery of common chemotherapeutics such as doxorubicin (DOX), platinum compounds cisplatin (CIS) and oxaliplatin (OXA), methrotrexate (MTX), sorafenib (SOR), and curcumin (Cur), with a focus on their advantages compared to chemotherapeutics alone. Targeted delivery of miRNA and siRNA will also be covered in a dedicated section. Theragnostic approaches for the simultaneous imaging and diagnosis of cancer will also be presented in a separate section, due to their promising perspectives in the field of cancer management.

## 2. Classification of BMNPs

In order to properly choose the most appropriate MNPs for specific biomedical applications, a very clear classification of them according to their nature, composition, and size is required, taking into account the synthesis methods used to prepare them and the specific functionalization of the surface.

**Figure 1:**
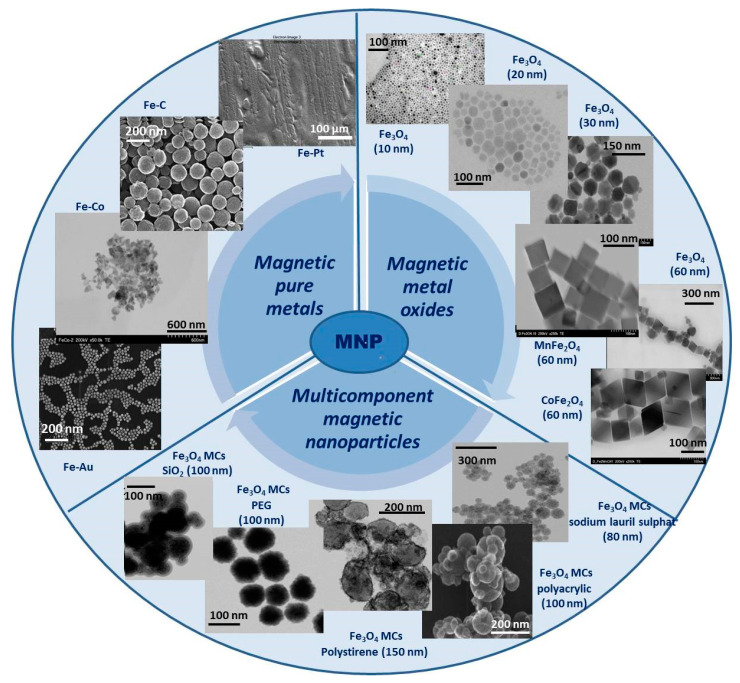
General classification and exemplification of magnetic nanoparticles (all images are original and belong to the authors).

Over time, several types of MNPs have been developed and researched. Generally, these MNPs can be grouped into three main categories as (i) magnetic pure metals (Fe, Co, Ni), (ii) magnetic metal oxides (Fe_2_O_3_, Fe_3_O_4_) or ferrites (MeFe_2_O_4_, Me = Fe, Co, Zn), and (iii) multicomponent magnetic nanoparticles as core/shell MNPs or magnetic nanoclusters (Figure 1). Each of these mentioned categories has both advantages and disadvantages, but their properties can be adapted to fit a particular type of application. In the following, each category will be discussed and exemplified.

### 2.1. Magnetic Pure Metals

Pure metal type materials exhibit some unique properties, with some of them directly dependent on the distribution of electrons in the external orbitals, and magnetic behavior is one of these properties. Transition metals, such as Fe, Ni, Co, and Mn, are the most commonly used in this class because they show good magnetic performance in several biomedical fields [39].

Iron (Fe) nanoparticles are one of the most common ferromagnetic materials used for biomedical applications. The main advantage of using this material is related to its excellent magnetic properties, which can be exploited in a wide range of biomedical applications.

In terms of synthesis methods, Fe nanoparticles can be obtained by relatively simple methods such as the reduction of iron salts in aqueous solutions in the presence of reducing agents such as sodium borohydride [40], or by thermal decomposition of Fe(CO)_5_ on a polymer matrix [41]. Even if the synthesis methods are facile and accessible, one of the major weaknesses of these NPs is that synthesis requires rigorous control of the surface-covering shell since direct contact between the Fe surface and air leads to their combustion. Therefore, the necessity to find homogeneous and uniform coatings for this kind of nanoparticle is critical. There are studies in which tailoring the reaction parameters and changing the Fe precursor to Fe[N(SiMe_3_)_2_]_2_ can result in major improvements in the reaction yield, better control of the size distribution, and a reduction in by-product formation [42].

In addition to Fe NPs, cobalt (Co) is another magnetic material commonly used in biomedical applications. Although its toxicity is higher than that of Fe NPs, beneficial effects of its use have been observed in specific MRI imaging applications and local hyperthermia of malignant tumors [43,44].

### 2.2. Magnetic Metal Oxides

From the general class of metal oxides, iron oxide distinguishes itself as a material with excellent magnetic properties and low toxicity compared to pure metallic forms, which, due to its unique physio-chemical properties, has potential applications in nanotechnology [39]. Over time, metal oxides have gained popularity in biomedical applications due to their physicochemical and mechanical stability, low toxicity, and biocompatibility but also for their efficiency in some special areas of biomedical science such as magnetic bio-separation of some species of interest, magnetic hyperthermia, drug carriers at target sites, and bio sensing [45,46].

There are three types of iron oxides: Iron (III) oxide, with two subspecies: Hematite (α-Fe_2_O_3_) and maghemite (γ-Fe_2_O_3_); a rare type of iron (II) oxide (FeO) called wüstite; and iron (II, III) oxide named magnetite (Fe_3_O_4_). This different stoichiometry due to the flexibility of the Fe oxidation state (Fe^2+^/Fe^3+^) is supported by the formation of different single-crystal phases with different chemical and physical properties.

Of all iron oxide structures, Fe_3_O_4_ has the most closely packed cubic inverted spinel structure and semi metallic properties, demonstrating great potential in biomedical fields [47]. One of the biggest advantages of these NPs is the ease of their synthesis at relatively high reaction yields by simple and conventional methods. In the beginning, conventional preparation methods such as the co-precipitation or solvothermal method [48,49,50,51,52,53] did not offer rigorous control over the size and size distribution of MNPs, so aggregation and poly dispersibility appeared, which hindered their use in biomedical applications. Over time, however, new synthesis methods were explored to obtain size-controlled NPs with a uniform size distribution. For example, by using organic iron precursors, Fe_3_O_4_ NPs with a narrow size distribution were obtained. By adjusting the molar ratio between metal precursors and different surfactant molecules, different particles can be obtained ranging in shape from cubic to polyhedral [54]. It can be concluded that the physicochemical properties of NPs, in particular the magnetic properties, can be adjusted according to the intended application with minimal changes in the composition and synthesis parameters.

Although iron oxides have the advantage of easy preparation, they present certain limitations related to chemical stability under biological environmental conditions, often resulting in aggregation phenomena that decrease their potential use in biomedical applications. Coating the surface of iron oxide NPs with biocompatible molecular layers increases chemical and mechanical stability, reduces surface oxidation, and decreases toxicity in biological entities. Thus, iron oxide NPs, generically called superparamagnetic iron oxide nanoparticles (SPIONs), have been developed. These particles represent small synthetic iron oxide particles with a core ranging between 10 nm and 30 nm in diameter, coated with certain biocompatible molecules, which provide chemical handles for the conjugation of therapeutic agents and improve their blood distribution profile [55]. These particles exhibit superparamagnetic properties, meaning that under an external magnetic field, they magnetize to saturation magnetization, and when the magnetic field is removed, they exhibit no residual magnetic interaction. This property is dependent on the size of the NPs and generally occurs when their size is only 10–20 nm. At such a small size, these NPs do not exhibit multiple magnetic domains, as found in larger NPs, acting as a “single super spin” that exhibits high magnetic susceptibility. Thus, upon application of a magnetic field, these NPs provide a stronger and faster magnetic response compared to bulk magnets with negligible residual magnetization and coercivity (the field required to bring the magnetism to zero) [56]. This unique property of SPIONs represents a great advantage for their use in specific biomedical applications such as controlled drug delivery where these NPs function as drug delivery vehicles because they can drag specific drug molecules to the target site, under the influence of an external magnetic field. Furthermore, once the applied magnetic field is removed, the magnetic particles do not retain any residual magnetism and aggregation phenomena are avoided, thus evading absorption by phagocytes and increasing half-life in circulation. In addition, due to their negligible tendency to agglomerate, SPIONs pose no danger of thrombosis or blockage of blood capillaries [55].

A sub-division of metal oxides, emerging from the necessity of adjusting the properties of this type of material for specific biomedical applications, is spinel ferrites (MeFe_2_O_4_), a very important magnetic material due to their combined electrical and magnetic properties, which make them useful in many technological applications [57]. The main component of these materials is iron oxide doped with a variety of bivalent transition metals such as zinc (Zn), manganese (Mn), cobalt (Co), nickel (Ni), iron (Fe), platinum (Pt), and palladium (Pd). It has been demonstrated that due to the doping of the Fe_3_O_4_ structure with bivalent cations, ferrites show improved electrical and magnetic properties for hyperthermia applications, for example, but also present the disadvantage of higher toxicity compared to magnetite [58]. The use of some stabilizing molecules, which also confer biocompatibility on the ferrite NPs surfaces, is essential for biomedical practical applications. In the synthesis of Co ferrite NPs, a CoFe_2_(CO)_9_ precursor and two stabilizing agents, hexadecylamine and oleic acid, were used to obtain small, relatively polydisperse MNPs. When a third stabilizing agent, lauric acid, was introduced, bigger monodispersed crystalline structures with a narrower size distribution were obtained. A decrease in the toxicity of the system was also demonstrated, with the developed system showing promising applications against tumor cells [59]. Another particularly efficient approach to using ferrite NPs in biomedical applications is their coating with biocompatible polymer layers, such as polyhydroxy and polyamine-type polymers and polyethylene glycol (PEG). It has been proven that the use of these systems in controlled drug release applications is particularly effective, with researchers also performing biocompatibility tests, which demonstrated that these materials can be successfully applied without adverse effects regarding toxicity [60]. Other ferrite-type magnetic structures have been reported in the literature, which, in addition to excellent magnetic properties, also show lower toxicity, such as Zn-, Mn-, and Ni-based ferrites [61]. There are also ferrite-type systems, with two transition metal components, Co-Zn and Mn-Zn type, in which it was highlighted that the concentration of Zn ions has an effect on the size of the final particles, i.e., increasing the concentration of Zn cations decreases the size of the particles [62].

### 2.3. Multicomponent Magnetic Nanoparticles

Multicomponent MNPs have been developed as a normal evolution of the technology because these systems can present multiple functionalities at the same time, by simply combining two or more components and thus offering new features that are not available in single-component materials or structures. In addition, in these multicomponent systems, improved specific properties can be obtained that may overcome the natural limitations of single-component materials. There are several types of multicomponent magnetic structures, but the most often studied and with real possibilities of application in biomedicine are the two main categories: (i) Core/shell type multicomponent MNPs and (ii) magnetic clusters (MCs).

(i) Core/shell-type multicomponent magnetic nanoparticles

Core/shell-type MNPs are the most common type of multicomponent NPs and have been widely studied. Core/shell structures were first prepared in semiconductor NPs [63] with the specific aim to modify their electrical and optical properties. The extension of this idea to magnetic systems initially emerged as an idea to protect and enhance the properties of metallic MNPs by coating them with protective layers, especially Fe NPs, which have extremely high reactivity in metallic form.

Core/shell composite structures of Fe/Fe_3_O_4_ type with a magnetic core consisting of iron NPs (Fe) and a thin protective coating of iron oxide (Fe_3_O_4_) were prepared [64]. Following the same idea of covering magnetic cores of other metals, various multicomponent magnetic systems have been developed, in which the metallic magnetic core was synthesized by the so-called seed-mediated process followed by coating with oxide layers of the same metal and/or various metallic combinations, such as Ni/NiO [65], Co/CoSe [66], Co/Co_2_P [67], and Pt/Fe_2_O_3_ [68]. Another successful possibility consisted of covering metallic Fe NPs with a ferrite shell (Fe@MFe_2_O_4_, M = Fe, Mn, Co) [69]. The core/shell composite system kept high-level magnetic properties, presented high relaxivity, and remained mono-dispersed throughout the experiments without showing aggregation processes under the action of the biological environment.

There are also core/shell type multicomponent magnetic systems in which the magnetic core is bimagnetic, obtained by an interesting combination of two magnetic metallic species, covered as a whole with an oxide coating. A successful example of such synthesized systems is the FePt/Fe_3_O_4_ core/shell system where MNPs from the core were obtained by sequential decomposition of some organic precursors Fe(acac)_3_ and Pt(acac)_2_ and the thickness of the external shell was tailored from 0.5 nm to 5 nm by controlling the synthesis parameters [70]. The seed-mediated growth method was extended to prepare other types of core/shell binary systems such as FePt/ZnO [71], Fe_3_O_4_/ZnO [72], Co/CdSe [73], FePt/PbS [74], and Fe_3_O_4_/Cu_2−x_S [75].

Another model of multicomponent core/shell systems, in which the magnetic core was made of iron oxide NPs (Fe_3_O_4_) covered with a noble metal coating, was also obtained. The as-prepared systems Fe_3_O_4_/Au [76] and Fe_3_O_4_/Au/Ag [77] were later functionalized appropriately for the intended biomedical application. This system not only benefits from the excellent magnetic properties of the SPION core but also from the plasmon resonance properties of the noble metals in the combination of the multicomponent magnetic system [78].

(ii) Magnetic clusters

In recent years, MC systems obtained by controlled self-assembly of superparamagnetic nanoparticles into three-dimensional clusters have gained popularity in biomedical applications, based on the new, innovative, collective properties that hold great promise for the development of advanced bio nanomaterials with novel integrated functions [79]. Depending on the preparation method and the nature of the functionalizing polymers, MNPs can be assembled with different spatial orientations resulting in clusters with different shapes and sizes (spheres, rod-like, nanoring, etc.) [80,81,82], which influence their properties. MCs with a polymer coating are very promising for theragnostic applications since they ensure an increase in the performance of MRI and hyperthermia and allow encapsulation of the drug that can be released at the target [80,81,83].

MCs are defined as systems consisting of a finite number of interacting spins ranging from a few hundred to several thousand, with well-controlled sizes, shapes, and compositions, which are magnetically isolated from the environment [84]. From the general definition of MCs, an important subclass of MCs is distinguished, consisting of several hundred MNPs with superparamagnetic properties [85,86], self-assembled as up to 100 nm MCs. In these systems, the spin dynamics exhibit many features similar to those of the molecular nanomagnets, such as superparamagnetic relaxation and quantized spin-wave states [87].

Since some of the properties of MCs depend on both the sizes of the MCs and the component NPs, considerable effort is required to control these two structural parameters in order to establish the optimal structure–property relationship and to obtain multicomponent magnetic systems with specific properties for biomedical applications. The development of robust synthesis protocols that allow rigorous control over the composition, size, and shape, as well as cluster surface properties and uniformity of colloidal NPs, requires a very good understanding of the mechanisms that induce aggregation phenomena. The forces involved in the controlled aggregation process of MNPs are both covalent and non-covalent such as hydrogen bonding, electrostatics, and van der Waals interactions, forces that can be controlled by the modification of synthesis parameters, such as the nature and concentration of solvents, the surfactants involved, the reaction temperatures, and reaction times [79].

The literature presents a wide variety of preparation methods for MCs, including options that involve several simple procedures integrated into a single reaction step or more complex versions that produce MC in a single reaction step. A classification of MC preparation methods can therefore be made as follows: (a) Single-step MC procedures, which integrate the synthesis of MNPs and their controlled aggregation into clusters in a single step, and (b) multi-step MC procedures, which first produce MNPs of a specific size, shape, and surface functionality and then self-assemble into clusters of required geometrical configurations in subsequent separate steps. Each of the synthesis methods has both advantages and disadvantages. While single-step processes are easier in terms of methods and more efficient in terms of producing MCs, the control of the synthesis parameters is less rigorous because the NPs’ synthesis and the clustering steps take place at the same time. Multi-step processes have the advantage of being more flexible in terms of primary materials, being able to produce a wide variety of materials in cluster form with well-organized structures, but they also bring a higher complexity, as well as some disadvantages in terms of magnetic properties, which may decrease due to the functionalization of the surface with relatively thick layers of non-magnetic materials.

(a) Single step-magnetic cluster procedures

From the most popular preparation techniques for the synthesis of MCs in a single reaction step, we can list the thermolysis method and the solvothermal method. In the following, some literature references of each of these methods will be exemplified, although it should be noted that there is no generally accepted method, and each synthesis of MCs with different shapes, sizes, structures, and surface functionality will involve specific synthesis parameters that cannot be reproduced for other versions.

The thermolysis method involves the reaction of metal precursors in the presence of the capping ligand in an organic solvent at a high temperature. Due to the high temperature, the metal precursor decomposes, and the nanoparticle seed and growing reaction is initiated. At this point, the capping ligands bind to the surface of the NPs, limit their growth, and prevent the formation of interparticle bonds, also leading to the clusterization of the NPs in controlled shape and size clusters through steric interactions between ligand molecules. The nature and concentration of the capping ligands play an essential role in obtaining structurally homogeneous clusters with a uniform size distribution. Using the thermolysis method, oxide clusters of transition metals such as In_2_O_3_, CoO, MnO, and ZnO were prepared by reducing the concentration of stabilizing organic ligands to the point where insufficiently stabilized primary NPs agglomerated in a controlled three-dimensional form [88]. The thermolysis method has also been successfully implemented to produce MCs with Fe_3_O_4_ as primary MNPs in a single reaction step at a high temperature to obtain polyelectrolyte-coated MCs [89]. In this process, basic hydrolysis of the iron precursor (FeCl_3_) using diethylene glycol as a solvent and polyacrylic acid as a surfactant took place at 220℃. The size of the MCs could be controlled by modifying the synthesis parameters, such as varying the pH and controlling the molar ratio between the metal precursor and surfactant, resulting in homogeneous geometry and narrow size distributions of MCs with sizes between 30 and 180 nm.

The solvothermal method consists of chemical reactions of metal precursors in a closed environment called and autoclave at high temperatures, higher than the boiling point of the solvent. This method has very quickly become a commonly used method due to its versatility and simplicity of application in terms of materials, methods, and cost. There are, however, several disadvantages of this method, namely the relatively high energy requirements, the reactions taking place at high temperatures for long reaction times, the limited scalability, and the lack of direct monitoring of the reaction process. In the literature, there are many studies that describe the preparation of MCs obtained by the solvothermal method [48,49,50,51,52,53].

(b) Multi-step magnetic cluster procedures

Multi-step MCs procedures involve the preliminary preparation of MNPs, using traditional methods that are subsequently assembled in one or more reaction steps in clusters with controlled shapes and sizes. This method offers the opportunity to combine the inherent properties of MNPs with innovative properties resulting from the collective interaction of NPs within the clusters. A large number of new composite materials commonly produced in the form of NPs with excellent control over size, shape, and surface properties can be developed easily and with remarkable properties, which can later be modularly assembled in the form of super-structured nanomaterials with various configurations and programmable properties.

One of the simplest methods to obtain MCs in two reaction steps was the encapsulation of the MNPs in different encapsulating agents with a specific function for controlled aggregation, protection, and surface functionalization. For the embedding process, different types of molecules were used as encapsulating agents, including polymers and/or block copolymers [90,91,92], micelles [93], polysaccharides [94], or hydrogels [95].

Another efficient clustering method is the emulsion method, slightly modified as the mini-emulsion method, if MCs of hundreds of nanometers in size are obtained. The mini-emulsion method can have two alternatives, direct emulsion, generically called “oil-in-water emulsion” in which oil (nonpolar solvent) droplets form micelles in an aqueous medium containing a water-soluble surfactant, or inverse emulsion, generically called “water-in-oil emulsion” in which water droplets form micelles in an organic medium with a non-polar dissolved surfactant. For the preparation of MCs, the mini-emulsion oil-in-water method is most commonly used because the pre-prepared magnetic particles can be easily dispersed in an organic solvent and used as the organic oil phase. In this process, MNPs prepared in a previous reaction step are homogeneously dispersed as a colloidal solution in a non-polar organic solvent, such as cyclohexane, dodecane, or toluene, to form an organic phase, generically called the “oil” phase. At the same time, a surfactant aqueous solution is prepared having dissolved a surfactant agent such as sodium dodecyl sulphate or cetyltrimethylammonium bromide, which has an amphiphilic structure. Using an ultrasonic finger, the two non-miscible phases are mixed to form a mini-emulsion. The mini-emulsion represents droplets of the organic phase, oil, containing the MNPs dispersed in water, where the surfactant is oriented with the hydrophobic end to the oil droplets and the hydrophilic end to the aqueous medium, creating stable oil in water micelles. The oil-in-water mini-emulsion is heated and the organic solvent from the micelles is evaporated, forming hydrophilic MCs. The size of these clusters can be adjusted by changing the concentration of NPs in the medium and the surfactant in the oil phase [96]. Using this method, composite clusters have been obtained by encapsulating various types of nanoparticles in different micelles, and/or molecular layers, polymers, and organic or inorganic layers such as γ-Fe_2_O_3_/TiO_2_ [97], NaYF_4_-Yb,Er/NaYF_4_:Eu [98], CeO_2_/Pd [99], polystyrene [100], and Fe_3_O_4_/OA-FeCo/Al_2_O_3_ MCs [101].

## 3. Limitations and Solutions to Overcome These Limitations in the Use of MNPs in Bio-Nano Medical Applications

**Figure 2:**
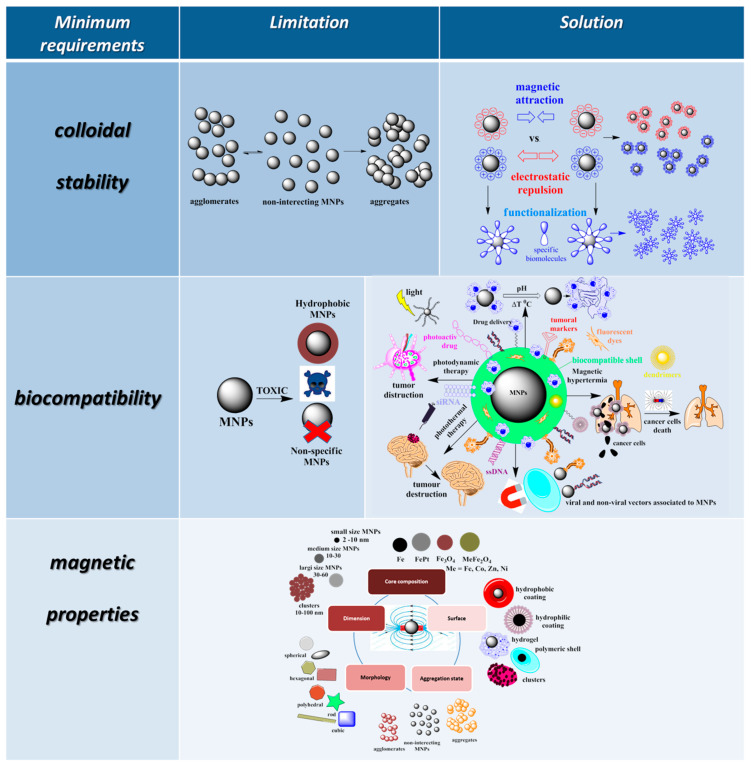
Strategies to bridge the gap between magnetic nanoparticles (MNPs) and bio-magnetic nanoparticles (BMNPs).

Over time, based on a deeper understanding of the action mechanism of bio-MNPs in biomedical applications, some ideal properties, considered minimum requirements for the functionalization of MNPs, were proposed for higher performance of these materials in practical nano-bio applications (Figure 2).

These minimum requirements have been established based on some limitations relative to the direct applications of these materials in bio-nano medicine. One of the most important is related to the colloidal stability of MNPs in the aqueous medium, when agglomeration and/or aggregation phenomena occur. Another limitation is the potential negative effects on the human body and the environment [18]. Proper surface functionalization increases the compatibility of MNPs with the biological environments, making them biocompatible and biospecific. Significant research efforts are dedicated to suitable surface modifications of magnetic nanoparticles to improve colloidal stability, prevent aggregation of nanoparticles, ensure a non-toxic state in physiological conditions, and introduce functional groups for binding of application-specific target molecules [7,14,15,18,19], thus the appropriate coating of NPs is very important to ensure their stability in physiological and biological fluids [18,19,102].

The first minimum requirement for the use of these NPs in biomedical applications is their surface functionalization to prevent MNPs from aggregation and lead to improved colloidal stability [103]. The colloidal stability of the MNPs can be improved by balancing the attractive and repulsive forces between these materials and their environment. Stabilization of MNPs can be performed by controlling the intensity of one or both repulsive forces, which are electrostatic repulsion and steric repulsion. Steric stabilization is based on coating MNPs with various molecules or a ligand shell or embedding them in an inorganic or polymeric matrix with steric properties. In these cases, the repulsive potential is given by the molecular weight and/or density of the surface coating material and its chemical and mechanical stability but also by the quality of the dispersion medium in which the functionalized magnetic material is used. The mechanism behind steric stabilization refers to the fact that if two sterically stabilized MNPs approach each other and exhibit steric properties, a connection is created between them that has the effect of reducing the entropy of the material, increasing the osmotic pressure between the functionalized MNPs, and leading to increased colloidal stability [104].

Another very important property necessary for the application of MNPs in biological environments is the compatibility of these NPs with the aqueous medium. An appropriate strategy to overcome the limitations imposed by the use of uncoated, hydrophobic, or weakly hydrophilic MNPs is crucial for the stability of MNPs in a biological environment and depends on the nature of the MNP surface. If MNPs are uncovered, the direct attachment of hydrophilic agents to the surface of uncoated MNPs in the final stage of MNP synthesis can be chosen as a compatibility strategy. If MNPs are already coated with a hydrophobic layer, the addition of amphiphilic molecules containing both a hydrophobic segment and a hydrophilic component can be used for water compatibility. In this case, the hydrophobic part forms a double-layer structure with the initial hydrophobic surface of the MNPs, while the hydrophilic groups are exposed on the outside of the NPs, which makes them water soluble. Another compatibility strategy when the surface of MNPs is hydrophobic or weakly hydrophilic is the ligand exchange method, with the direct replacement of the original surfactant with a new one, which can improve the hydrophilic properties of MNPs [105].

Another particularly important requirement when it comes to the use MNPs in bio-medical applications is the magnetic properties of these materials, which depend on their nature and size. In applications where these NPs involve exposure to an external magnetic field, their shape and size, as well as magnetic properties such as their superparamagnetism and saturated magnetization value, are essential criteria for material selection. Very small-sized particles, below 10 nm, despite presenting colloidal and magnetic stability, are very difficult to process and have relatively low magnetization values. Therefore, slightly larger NPs are preferred because they are easier to manipulate magnetically, have higher magnetization values, and at the same time, have lower colloidal stability. A new and original alternative that overcomes the limitations of colloidal and magnetic instability while having superparamagnetic properties and high saturation magnetization values is represented by MCs, which are self-assembled, controlled aggregations of hundreds of single-core MNPs in the form of spherical clusters of approximately 100 nm in size.

One of the particularly important requirements for using MNPs in biomedical applications is low toxicity to the biological environment and biocompatibility of the material. It is known that, due to their large surface area and chemical reactivity, MNPs can generate reactive oxygen species that can penetrate tissues down to the cellular level, thus being extremely toxic [106,107]. MNPs can exert their toxic activity at multiple levels, leading to potential mitochondrial destruction, DNA damage, temporary or permanent cell cycle cessation, or cell membrane disruption [108]. It is known that the shape and size of MNPs, the hydrophilicity of the surface, the mass/thickness of the surface covering material, as well as the surface charge are key factors governing the biocompatibility of the material. An important strategy for improving biocompatibility and reducing toxicity is represented by the functionalization of MNPs with different agents, such as polymers, proteins, or inorganic compounds. Proteins that are abundant in human serum, such as albumin, can create a protective barrier on the surface of MNPs, increasing biocompatibility [109]. Coatings with silica, which is abundant in hydroxyl groups, also generate less-toxic MNPs [110]. Some studies suggest that coating with noble metals such as gold can also increase biocompatibility [111]. Biocompatible molecular layers covering MNPs with negative charges show better biocompatibility, while a positively charged shell on the surface shows better cooperation with bio systems but can often interact electrostatically with differently charged particles of a biological medium. Biocompatible polymeric coatings represent a useful alternative because they can decrease the density of the material, making them more stable from colloidal and magnetic points of view and, at the same time, offer advantages on further surface functionalization with different biologically active substances for specific applications. From the synthesis method point of view, MNP surface functionalization with positively or negatively charged molecular layers is easier and much more controllable, while surface coatings with polymeric layers offer much less control over the morphology of the final material, resulting in relatively thick polymeric layers and therefore slightly lower magnetic properties.

## 4. Considerations about the Development of Magnetic Drug Delivery Systems

The main goal of DDSs is to ensure that the chemotherapeutic drug reaches its intended site of action in the body, that is, the tumor and cancer cells, while minimizing its distribution to healthy cells, reducing side effects and increasing therapeutic efficacy.

There are several types of DSSs [112,113,114]:

(i) Oral drug delivery—the most common form of drug delivery, where the drug is taken orally in the form of pills, capsules, or liquids. In this case, the drug is absorbed into the bloodstream through the digestive system.
(ii) Injectable drug delivery—a method that involves injecting the drug directly into the bloodstream, intramuscularly or subcutaneously. This type of delivery is used for drugs that cannot be taken orally or that need to be delivered quickly.
(iii) Topical drug delivery—a method of delivery that involves applying the drug directly to the skin, mucous membranes, or other external surfaces of the body. Pharmaceutical formulations such as creams or ointments are commonly used in this case.
(iv) Inhalatory drug delivery—a method that involves delivering drugs through inhalation into the lungs. This type of delivery is commonly used for the treatment of respiratory diseases.
(v) Implantable drug delivery—a method of delivery that involves implanting a device or a drug-eluting implant that slowly releases the drug over a period of time. This method is commonly used for long-term treatments.

In addition to these traditional DDSs, there are also newer technologies such as targeted drug delivery, where drugs are delivered specifically to cancer cells, and nanotechnology-based drug delivery, where drugs are delivered using NPs, technologies that are still in development but hold promise for the future of drug delivery [115,116].

MNPs have drawn considerable attention in recent years due to properties that make them promising candidates for drug delivery, with numerous studies published in this field. In the successful development of magnetic DDSs, some considerations about their physical properties, targeting abilities, cargo release, and cytotoxic properties need to be taken into account.

In general, iron oxide NPs, especially magnetite, are used for the development of magnetic DDSs. The most important properties of these carriers are their size and superparamagnetism. In order to be used for in vivo applications, the size of DDSs needs to be between 10 and 100 nm in diameter [7,8]. Particles that are smaller in size are quickly eliminated by the kidneys and cannot perform their biological role in vivo, while bigger particles are recognized by the immune system and are trapped in the reticuloendothelial system [117,118]. The size of dehydrated magnetic NPs is determined experimentally using electron microscopy techniques such as transmission electron microscopy (TEM) or scanning electron microscopy. Another characteristic is the hydrodynamic size, which is the size of the hydrated NPs in an aqueous solution and is generally determined by dynamic light scattering [119].

Biomedical applications generally focus not on simple MNPs but on magnetic nanocomposites that combine the properties of MNPs with those of other components, leading to complex properties such as increased biocompatibility and stability, low toxicity, and high drug-binding capacity. The shell of these magnetic nanocomposites can be represented by polymers (PEG, chitosan, carboxymethyl chitosan, poly(methacrylic acid), polydopamine, poly(lactide-co-glycolide), polyvinyl alcohol, polyethyleneimine), mesoporous silica, layered double hydroxides, liposomes, sugars, proteins, inorganic compounds, or lipids. The successful functionalization can be confirmed by Fourier Transform Infrared Spectroscopy or Energy Dispersive Spectroscopy.

Drugs are incorporated into magnetic DDSs by interacting with the shell, either by covalent binding or electrostatic interactions. The loaded DDSs can be characterized by two parameters, namely drug-loading capacity (LC) and drug encapsulation efficiency (EE). The LC represents the amount of drug that is loaded in 100 g of DDS and helps determine the amount of drug that could be delivered by the DDS to its target. EE represents the percentage of drug that was loaded into the DDS from the feeding solution [120].

Once the DDS is obtained and loaded with the desired drug, it needs to be delivered to the target organ. In the treatment of cancer, targeting can be performed in either a passive or an active manner. Passive targeting relies on the EPR effect, which allows preferential accumulation of nano-DDSs in the tumor tissue due to increased permeability of blood vessels and retention at the tumor level due to low lymphatic drainage. However, EPR offers only a modest increase in accumulation at the tumor level compared to healthy organs, so active targeting was developed as an alternative [117,121]. Active targeting can be achieved by the functionalization of the magnetic DDSs with ligands that can specifically bind to structures present on the surface of cancer cells. Examples of such ligands are antibodies, peptides, proteins, aptamers, folic acid (FA), or hyaluronic acid (HA) [121]. In the case of magnetic DDSs, targeting can be aided by the application of an external magnetic field that guides the DDS to the desired location.

Once at the delivery site, the DDS needs to be able to release the loaded cargo. In the treatment of cancer, the special properties of the tumor microenvironment are used to preferentially deliver the drugs to the tumor. Most DDSs investigated in this review present a pH-dependent release behavior, which relies on the lower pH of the tumor microenvironment compared to that of healthy cells or blood [122]. Other examples of preferential release include the use of substrates that are degraded by enzymes overexpressed at the tumor site [123,124,125,126,127], redox-dependent release based on the higher concentration of glutathione (GSH) in tumor cells compared to healthy ones [128,129], or magnetic-field-mediated release [128,130,131,132,133,134,135,136,137].

Ideally, drugs encapsulated in DDSs should present higher toxicity towards tumors and lower toxicity towards healthy organs compared to the free drug. Toxicity assessments were generally carried out on cell cultures, while few studies also focused on the in vivo effects of the DDSs using animal models.

## 5. Chemotherapy Delivery Using Magnetic Nanoparticles

Chemotherapy represents one of the mainstays of cancer treatment. Its primary goal is to stop cancer cell proliferation and the formation of metastases [3]. There are different classes of chemotherapeutic drugs, based on their primary mechanism of action: Alkylating agents, antimetabolites, antimicrotubule agents, topoisomerase inhibitors, antibiotics, and others [138]. Despite having different mechanisms, all chemotherapeutics present toxicity towards malignant cells but also towards healthy cells, producing side effects such as myelosuppression, hair loss, nausea, and vomiting. In recent years, efforts have been made to improve the selectivity of chemotherapeutics for cancer cells and tumors by using different strategies. One such strategy is the incorporation of chemotherapeutic drugs into DDSs. DDSs offer several advantages over classical chemotherapy treatment [115,116,139,140]:

- One of the main advantages of DDSs is the ability to target specific cells or tissues, minimizing damage to healthy cells.
- DSS use reduces side effects associated with chemotherapy, because DDSs deliver drugs directly to the site of action.
- DDSs can improve the efficacy of drugs by ensuring that they are delivered to the intended site of action and in the correct dosage.
- Some DDS technologies, such as implantable drug delivery devices, can release drugs slowly over an extended period, ensuring sustained and controlled release of the drug.
- DDSs can be personalized to the individual patient, taking into account factors such as age, weight, and the specific characteristics of their cancer. This can help to ensure that the patient receives the optimal treatment for their individual case.
- DDSs can help reduce the development of drug resistance.

Overall, DDSs have the potential to provide more effective, targeted, and personalized treatments for cancer patients, while reducing the side effects and toxicity associated with classical chemotherapy.

In this section, examples of magnetic DDSs will be discussed and presented comparatively. The examples are classified based on the drug they incorporate. An overview of the main characteristics of the incorporated drugs is presented in Table 1, while examples of DDSs for each drug are presented comparatively in Table 2.

### 5.1. Doxorubicin

Doxorubicin (DOX) is one of the most commonly used drugs in the treatment of a variety of cancers. Many attempts have been made to reduce DOX toxicity, with its incorporation into numerous DDSs. Indeed, DOX has become a “model” molecule for the development of magnetic DDSs, with many proof-of-concept formulations using this molecule.

MNPs functionalized with different composites have been intensively used for the development of nanocarriers for DOX. Numerous recent studies present different approaches regarding the synthesis, functionalization, drug loading, delivery method, and release of DOX. In order for DDSs to be effective in targeted therapy, it is crucial to improve their biocompatibility and targeting ability. To improve biocompatibility, various materials such as biodegradable polymers [130,145,146], sugars [129,130,147], lipids [133], and proteins [148,149] have been used. These materials can be used for coating purposes to reduce the toxicity and immunogenicity of the resulting DDS.

Regarding their indication, DOX-containing DDSs were developed for a wide variety of cancers, including breast cancer [123,147,150], liver cancer [131,133,145], lung cancer [151], and colorectal carcinoma [129,132,152].

A polymer coating often used in the development of DDSs is PEG, which has proven to be suitable both for the efficient loading of different drugs and with good properties in terms of biocompatibility. Thus, MNPs and boron nanosheets coated with a pH-responsive PEG polymer were used for the fabrication of a new DDS for which highly controlled loading and release processes were optimized for DOX. Both in vitro and in vivo experiments have demonstrated that the DDS can inhibit tumor growth, induce cancer cell apoptosis, and reduce the toxic effects of DOX, a potential alternative for liver cancer therapy [145]. Polysaccharides such as chitosan or its modified forms can also be employed for the functionalization of MNPs for DOX delivery. A study reported a nanocomposite material that combines two different components, GSH and magnetic-sensitive iron oxide NPs, which provide redox-responsiveness and magnetic sensitivity, respectively. The MePEG-grafted water-soluble chitosan backbone helps achieve biocompatibility and stability of the nanocomposites. The conjugation of DOX with chitosan via a disulfide linkage ensures controlled drug release in the presence of GSH, which is often found in higher concentrations in cancer cells. In vitro experiments showed that an external magnetic stimulus helps concentrate the nanocomposites and provides efficient internalization of the nanocomposites into cancer cells [129]. The use of aminated lignosulfonate and carboxymethyl chitosan as biopolymers for DDS fabrication also offers several advantages, such as biocompatibility and pH responsiveness. The optimized NPs allowed for the encapsulation, targeted delivery, and controlled release of DOX to cancer cells. The drug-loaded particles were tested on cancer cells using MTT assay and live/dead staining experiments, and both methods showed that the drug-loaded particles had a significant inhibitory effect on the growth of cancer cells [147]. Chitosan-functionalized ferrogels were fabricated, and the resulting nanostructure possesses unique characteristics such as high porosity, ultra-low density, and superparamagnetism with high DOX loading efficiency. The ferrogels also exhibit tumor-specific pH-responsive swelling, excellent biodegradation, remotely switchable drug release, high magnetic hyperthermia potential, excellent biocompatibility, and promising cell–material interactions as substantiated by MTT assay, cytoskeleton staining, and confocal imaging [130].

An innovative DDS based on dumbbell-shaped NPs of CoFe_2_O_4_/MoO_2_ was developed for liver cancer treatment using external magnetic and photothermal effects. These NPs were modified with poly(methacrylic acid), loaded with DOX, and sealed with phase-change material to prevent premature drug release. The cytotoxicity of the composite was evaluated on human liver tumor cells and the results showed that the combined procedure led to the highest killing rate of tumor cells, indicating the potential of the developed system for liver cancer treatment [131]. Magnetic nanocomposites coated with nylon-6 were also reported for the delivery of DOX. The composites were synthesized by the co-precipitation method and coated with biodegradable nylon-6 to increase biocompatibility. The magnetic composites demonstrated good loading capacity and pH-dependent release behavior. Cytotoxicity studies were performed on lung cancer cell lines and showed increased toxicity of the DDS compared to free DOX [153].

A drug-loaded magnetic microrobot that can polarize macrophages into the antitumor phenotype to target and inhibit cancer cells was developed. The in vitro tests demonstrated that the microrobots have good biocompatibility with normal cells and immune cells. DOX was loaded onto the surface of microrobots through electrostatic interactions and exhibited pH-responsive release behavior. The microrobots were able to target and kill cancer cells in a 3D tumor spheroid culture assay through dual targeting from magnetic guidance and M1 macrophages. The findings suggest that the combined use of magnetic control, macrophages, and pH-responsive drug release can improve the tumor-targeting and antitumor abilities of microrobots [151]. However, the size of the microrobots exceeds the nanometer range, indicating the possible uptake by the immune system in the case of in vivo administration. A pH-responsive mesoporous Fe_2_O_3_-Au-based DDS with magnetic targeting was also designed. The Fe_2_O_3_ particles were constructed with external mesopores and internal hollow structures, while Au NPs were connected on the surface of Fe_2_O_3_ through pH-responsive bonds for drug encapsulation [146]. Magnetic mesoporous silica NPs were developed as a multipurpose nanoplatform for delivering DOX and providing magnetothermal therapy to cancer cells. The core-shell-type particles were characterized for their magnetic and thermal properties, drug loading and release kinetics, and cytotoxicity against cancer cells. The results revealed high magnetic saturation and heating efficiency, efficient DOX loading and release, and significant cytotoxicity against cancer cells both in vitro and in vivo [128]. Another approach for the delivery of DOX consisted of obtaining mesoporous silica nanoparticles, which were then modified by growing magnetic NPs on their exterior. This nanocomposite was loaded with DOX and release studies were performed in different pH and temperature conditions. The results indicated that the release is both pH and temperature dependent [154]. Amino acids were also employed for MNP functionalization in the delivery of DOX. Cysteine-modified magnetite NPs loaded with DOX were extensively characterized. The release behavior proved to be pH dependent, with twice as much DOX released at acidic pH compared to neutral pH. However, the acidic pH value tested, pH 3, is lower compared to that of the tumor microenvironment (5–6). The NPs’ toxicity towards melanoma cells was investigated using cytotoxicity and apoptosis assays [155]. Another nanocomposite for DOX delivery was developed from magnetite NPs covered with calcium carbonate by ion co-precipitation. The nanocomposite demonstrated a high DOX loading capacity and pH-dependent release. Cytotoxicity studies carried out on cervical and breast cancer cells proved the high anti-cancer efficiency of the synthesized composites [156].

**Figure 3:**
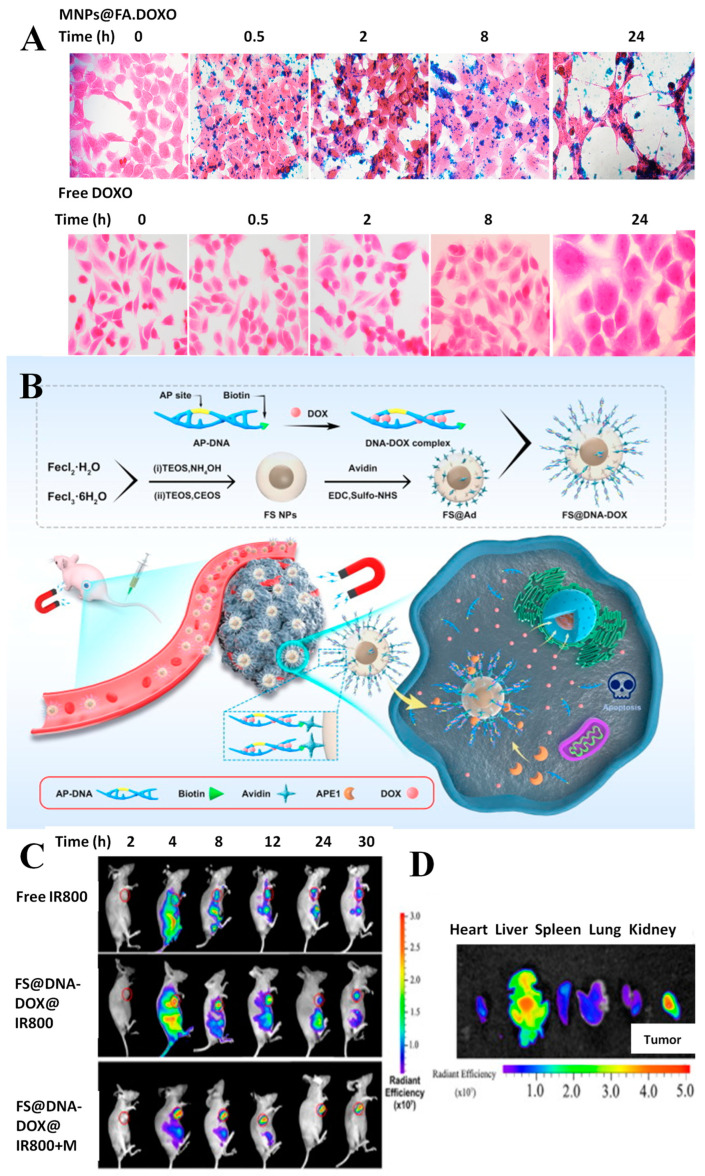
Analysis of (**A**) MNPs uptake and (**B**) cell morphology. (**A**) HCT116 cells treated with 1 μM MNPs@FA.DOXO. The intracellular iron from MNPs was evidenced by the Prussian blue assay. (**B**) HCT116 cells treated with free drug. The images are representative of three independent experiments. Cell staining: H&E. Magnification 400×. Reprinted with permission from Ref. [132] Copyright 2023 Elsevier; (**B**) The therapeutic mechanism of FS@DNA-DOX NPs as a magnetic-targeted and APE1-triggered drug delivery nanosystem. (**C**) Fluorescence images of mice after injection with IR800 and FS@IR800 NPs with or without magnetism. The red circles in the image represent the injection site. (**D**) The representative fluorescence images of major organs at 30 h postinjection of FS@DNA-DOX@IR800 with magnetism. Reprinted with permission from Ref. [123] Copyright 2023 Elsevier.

The development and characterization of an active targeting DDS were presented. Iron oxide NPs were functionalized with FA and loaded with DOX. The results indicated the successful loading and release of DOX combined with the efficiency of the nanosystems as selective contrast agents in MRI, with the data representing an initial stage in the validation of the nanotheranostic system before in vivo evaluation [157]. FA functionalization of magnetic NPs was also reported by Gentili et al. who developed a system for DOX delivery (MNP@FA.DOX). The administration of DOX using this system resulted in an enhancement of cell death compared to the free drug, confirmed by apoptosis assay. The images observed in Figure 3A correspond to Prussian blue staining of HCT116 cells treated with MNPs (up) and free DOX (down). The images show, as expected, the absence of precipitated iron in the cells that were not treated with MNPs@FA.DOX. In the case of delivering the drug by MNPs (up), it can be seen that the amount of intracellular iron rises and HCT116 cell size increased after 8 h of NP treatment. Figure 3B (down) shows the increase in the total size of the HCT116 cells and a marked increase in the dimensions of the cell nuclei, but no atypical morphological changes were observed for DOX treatment [132].

The interaction of magnetic DDS with cellular receptors is important and has been exploited in numerous studies. A magnetic liposomal carrier was used and showed targeted delivery of DOX to cancer cells via the αvβ3-integrin receptor, higher cytotoxicity in various cancer cell lines, and minimal toxicity to the heart tissue. This lipidic formulation was also found to have higher tumor accumulation and therapeutic efficacy compared to the clinical liposomal formulation of DOX (Lippod™), especially in combination with gamma radiation or magnetic hyperthermia therapy. The mechanism of chemo-radio-sensitization involved the activation of JNK-mediated pro-apoptotic signaling axis and delayed repair of DNA double-strand breaks [133]. Tannic acid-modified magnetic hydrotalcite-based MgAl NPs were synthesized for the targeted delivery of DOX to estrogen-receptor-expressing colorectal cancer cells. The in vitro release showed a pH-dependent biphasic and sustained release of DOX, while the safety and biocompatibility of the DDS were confirmed using hemolysis and MTT assays. Furthermore, higher cellular uptake in ER-positive HCT116 and LoVo cells was observed [152]. The development of a magnetic dual-responsive nanocarrier for targeted drug delivery to cancer cells overexpressing the DNA repair enzyme APE1 was described (Figure 3B).

The carrier consists of Fe_3_O_4_@SiO_2_ NPs and a specially designed AP-DNA strand that can be hydrolyzed by APE1. DOX was loaded onto the AP-DNA to form the theragnostic nanosystem (FS@DNA-DOX). The distribution of FS@DNA-DOX NPs was evaluated by in vivo fluorescence imaging and it was observed that after magnetic attraction near the tumor, the fluorescence intensity in the tumor location was significantly higher than that in other tissues, but without magnetic attraction, a strong distribution in the other organs was observed (Figure 3C). The fluorescence intensity per unit area in the tumor area was significantly higher than that in other organs after treatment with NPs with magnetism (Figure 3D) [123]. Some cancer treatment regimens involve drug combinations in order to enhance the treatment’s effectiveness. Thus, the encapsulation of different drugs in DDS is of interest for clinical practice. A novel approach, based on a core/double shells magnetic-luminescent nanomaterial was reported as nanocarrier for both MTX and DOX for simultaneous administration in breast cancer therapy. The encapsulation of the two drugs has been demonstrated with good efficiency, and a controlled pH-dependent drug release was observed. The encapsulation system has proven effective in cell line tests, being able to distinguish between normal and cancer tissues and cytotoxicity studies against MCF-7 cells demonstrated superior tumor inhibition compared to the free drugs [150].

### 5.2. Platinum Compounds

Magnetic DDSs were developed for the passive or active delivery of CIS and OXA. In most cases, magnetite was used as a core, and polymers or mesoporous silica were used to cover the magnetic cores. The release mechanism was generally pH-dependent.

**Figure 4:**
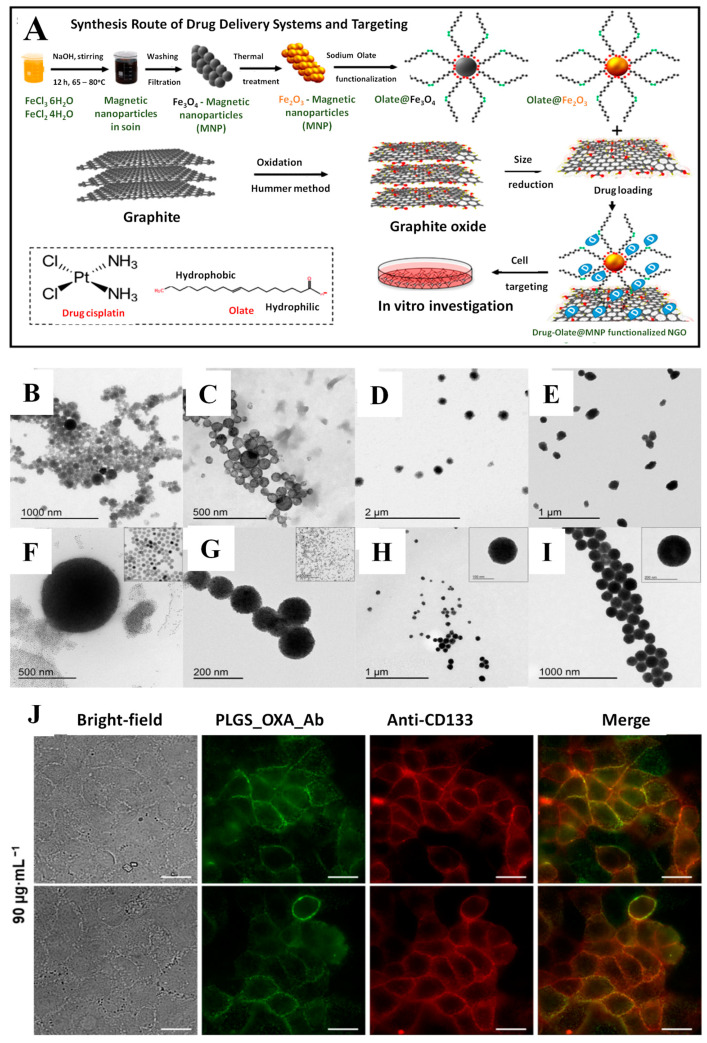
(**A**) Schematic representation of the synthesis process of maghemite NPs functionalized with nano graphene oxide for CIS-targeted delivery. Reprinted from Ref. [159]. (Open access); TEM images of Magnetic Nanoclusters synthesized using (**B**) low concentration of SDS surfactant; (**C**) low concentration of SPIONs; (**D**) SPIONs dispersed in THF; (**E**) SPIONs dispersed in hexane; (**F**) SPIONs with size of 12 nm; (**G**) SPIONs with size of 7 nm; (**H**) scaled up one-fold; (**I**) scaled up five-fold. Insets show SPIONs with size of (**F**) 7 nm and (**G**) 12 nm, low-magnification (**H**) MNCs scaled up one-fold and (**I**) MNCs scaled up five-fold. Reprinted with permission from Ref. [161]. Copyright 2023 American Chemical Society. (**J**) Fluorescence microscopy images of human cells derived from colorectal carcinoma (CaCo-2) treated with oxaliplatin-containing PLGA nanoparticles coated with anti-CD133 antibody conjugated to Alexa Fluor 488 (PLGA_OXA_Ab) and co-stained with antiCD133 Atto 565. The CaCo-2 cells were treated with 90 µg·mL^−1^ concentration of the PLGA_OXA_Ab for 30 min. From left: Bright-field images of the cells; fluorescence emission of cells treated with PLGA_OXA_Ab; cells stained with anti-CD133-Atto 565; merge of the fluorescence images. The scale bars correspond to 20 µm. Reprinted from Ref. [119] (Open access).

Magnetic NPs were functionalized with PEG and an ionic liquid for the delivery of CIS. The DDS demonstrated high platinum loading and stability in diluted human serum. The release was performed in simulated cytosolic media, which contains additives that make it more similar to real-life release conditions. However, no cytotoxicity tests were performed for the DDS [158]. Maghemite NPs were functionalized with nano graphene oxide (NGO) and the synthesis process is represented schematically in Figure 4A. The strong interactions between NGO and the CIS led to a slow-release profile, which followed reversible second-order kinetics. However, the release behavior was only tested at pH 7.4, with no tests carried out at the lower pH of the tumor microenvironment. The DDS exhibited in vitro toxicity towards U87 glioblastoma cells and the application of a magnetic field led to selective cell death in the area of magnet application [159]. Magnetite NPs covered in a silica layer and modified with dicarboxylic acid groups were developed as a potential treatment for pancreatic cancer. The DDS exhibited prolonged release at pH 7.4, with approximately 80% of loaded CIS released after 250 h. Cytotoxicity studies carried out on healthy cells and two pancreatic cell lines demonstrated selective toxicity towards cancer cells, with increased toxicity towards the highly metastatic line AspC-1 [160]. Rinaldi et al. reported polydopamine-modified MCs for the delivery of CIS. The clusters present better magnetic properties compared to magnetic NPs and can be guided more easily to the tumor tissue. The synthesis procedure was carefully optimized with respect to the surfactant, the solvent used, and the concentration of the nanoclusters, and the particles were characterized by TEM (results presented in Figure 4B–I). The resulting DDS showed pH-dependent release. The cytotoxicity of the DSS was tested on MCF-7 breast cancer cells and HeLa cervical cancer cells and demonstrated superior toxicity towards HeLa cells. The use of a magnet for the guidance of the clusters in cell cultures led to superior cytotoxic properties compared to the “no-magnet” approach, indicating the advantages of magnetic guidance [161].

Silica-coated magnetic NPs were also used for the active targeting of CIS, using HA as the targeting ligand. The developed system exhibited pH-dependent release, which followed Higuchi model kinetics. The DDS proved to be selectively toxic towards CT26 colorectal cancer cells compared to embryonic kidney cells and its cytotoxicity was higher compared to that of free CIS in cancer cells. In vivo experiments demonstrated that both the half-life and the area under the curve for CIS were increased when it was incorporated into the DDS, compared to CIS alone. Moreover, histological analyses revealed no toxic effects of the DDS on the kidneys, hearts, and lungs of tested rats [162]. Xie’s group also developed active targeting methods for the delivery of CIS, using either peptides [118] or FA [163]. In both cases, the toxicity of the constructed DDSs was higher compared to that of CIS alone in HNE-1 nasopharyngeal carcinoma cells, due to increased cellular uptake.

A passive OXA delivery approach was proposed by Darroudi et al. who developed chitosan functionalized magnetite NPs that displayed toxicity towards CT-26 colon cancer cells. However, more studies need to be performed, since the release behavior was not studied and the toxicity was not compared to that of free OXA [164]. Another passive approach involved the use of biomimetic magnetite NPs loaded with OXA. The release of OXA from these carriers followed a pH-dependent model. Moreover, upon application of a magnetic field, the release improved due to the hyperthermia effect, leading to a total release of 80% after 2.5 h. The DDS exhibited higher cytotoxicity compared to free OXA in T-84, HT-29, SW480, HCT-15, and MC-38 colon cancer cell lines [165].

Antibodies were used for the active targeting of OXA towards colorectal [119] and gastric cancer cells, respectively [166]. Magnetite particles were coated with poly(lactide-co-glycolide) and an anti-CD33 antibody was conjugated to the DDS. The DDS achieved a slow-release profile of OXA at pH 7.4, and its successful internalization in CaCo-2 cells via receptor-mediated endocytosis was demonstrated by fluorescence, indicating the successful co-localization of NPs and OXA in cancer cells (Figure 4J) [119]. Another antibody, Herceptin, was conjugated to iron-gold NPs for the targeted delivery of OXA. The DDS exhibited pH-dependent drug release and successful cell internalization. Moreover, animal studies demonstrated the specific accumulation of the DDS in tumor masses, indicating the selectivity of the DDS and its capacity to be used as a targeted delivery device.

### 5.3. Methotrexate

Several attempts have been made at encapsulating MTX into magnetic DDSs. Some of these used magnetite NPs functionalized with amino acids such as *L*-lysine (Fe-Lys) [124], arginine (Fe-Arg) [125], glycine (Fe-Gly) [126], or proteins such as bovine serum albumin (Fe-BSA) [127] in order to covalently bind MTX. The amino groups of the amino acids/proteins were covalently bound to the carboxyl groups of MTX to form an amide bond. Among these DDSs, Fe-Arg presented the smallest diameter, while Fe-BSA was the largest in size. The highest MTX loading efficiency was observed in the case of Fe-Lys. In all cases, the release of MTX from the carriers was dependent on the presence of proteinases, due to their capacity to cleave the amide bond in lysosomal conditions (acid pH). For the amino-acid-modified DDSs, the release of MTX took place in less than 12 h, while for the BSA-modified DDS, the release was slower. In the case of Fe-Gly, the release behavior was also pH-dependent and followed a Michaelis–Menten model, fitting an enzyme-dependent kinetics profile. Fe-Arg, Fe-Gly, and Fe-BSA demonstrated hemocompatibility in the hemolysis assay, while Fe-Arg and Fe-Gly also demonstrated biocompatibility towards fibroblasts. Fe-Gly and Fe-Arg demonstrated higher cytotoxicity towards breast cancer MCF-7 cells compared to MTX alone, while Fe-Lys and Fe-BSA demonstrated similar cytotoxicity compared to MTX alone.

Polymers and cyclodextrins have also been used to develop magnetic DDSs for the delivery of MTX. Magnetite superparamagnetic particles were covered with poly 3-hydroxybutyrate-co-3-hydroxyvalerate using a method optimized by the Box–Behnken design. The average size of the DDS was around 90 nm; however, large variations were noticed, with some particles exceeding 200 nm in diameter, which could be too large for EPR entrapment. The release of MTX at pH 6.8 from the carriers was slower compared to the release of free MTX in the same conditions, indicating a slow-release behavior. The toxicity of the DDS was higher compared to free MTX on SW-480 colorectal cancer cells [167]. Cationic polymers and cyclodextrins were also used as functionalizing agents for magnetite-based DDSs. MTX was loaded due to the electrostatic interactions between the cationic polymer and the negative charges of MTX at neutral pH. At the acidic pH of the tumor environment, the MTX was protonated, and the interactions were lost, leading to the pH-dependent release of MTX. The MTX was quickly released from the DDS and was successfully internalized in the cytoplasm of Saos-2 osteosarcoma cells. The cytotoxicity of the DDS was marginally higher than that of free MTX [168].

Active targeting was also employed for MTX delivery, using HA [169] or FA antibodies [170] as targeting agents. The HA-modified DDS displayed high drug encapsulation efficiency and the drug release was pH-dependent, with a higher than 10-fold increase in released MTX at pH 5.5 compared to pH 7.4. The DDS proved to be cytotoxic towards A549 lung cancer cells, with IC_50_ values much lower compared to those of MTX alone. Moreover, the DDS caused an increase in the expression of pro-apoptotic genes in cancer cells and a decrease in the expression of the gene encoding the pro-inflammatory cytokine tumor necrosis factor α (TNF-α). This demonstrates that the DDS has potential anticancer activity in A549 lung cancer cells, a type of cell that has high resistance to MTX [169]. Antibodies against folate receptors were synthesized and conjugated to MTX-modified magnetic NPs. The resulting DDS had high encapsulation efficiency for MTX and high binding efficiency for the antibody. The release of MTX was pH dependent and the DDS exhibited higher toxicity towards HeLa cervical cancer cells compared to MTX alone. Immunohistochemistry demonstrated the attachment of the DDS to FA receptors in mouse tumor tissue, and fluorescence analysis revealed the internalization of the DDS in cancer cells by endocytosis [170].

### 5.4. Curcumin

Curcumin (Cur) is a natural compound extracted from *Curcuma longa* and has attracted attention as a possible anticancer agent. Some studies have suggested that curcumin can help reduce the side effects of chemotherapy when used in association with it and can also improve patient survival due to its anti-oxidant and anti-angiogenic effects [171,172]. Despite its good safety profile and promising preliminary results, Cur has very poor bioavailability and water solubility, making it difficult to formulate and administer [172]. Different liposomal formulations have been fabricated to increase their bioavailability [172], and many proof-of-concept studies have associated Cur with magnetic NPs for both passive and active targeted delivery.

A passive targeted delivery approach used magnetite NPs covered with a layered double hydroxide (LDH), loaded with Cur, and finally covered with polydopamine [173]. The DDS demonstrated pH-dependent release in vitro and the polydopamine coating degraded progressively, allowing a controlled release of 63% of the drug after a period of 28 h. The DDS had lower cytotoxicity towards HepG2 liver cancer cells compared to Cur alone at the same concentration due to the time it took for the uptake, cleavage, and release of the drug from the DDS. However, cellular uptake studies demonstrated that Cur from the DDS could be efficiently internalized into HepG2 cells by endocytosis [173]. The same group also proposed an active targeting strategy for Cur, using polymer-modified magnetite NPs. The two monomers used were PEG methyl ether methacrylate and 4-vinylphenylboronic acid (VB), with the latter acting as both a site for the covalent binding of Cur and a ligand for the overexpressed sialic acid residues on the surface of cancer cells. The DDS showed a lower drug-loading capacity compared to the previous approach and pH-dependent release. Due to the stability of the boronate ester bond between Cur and VB at neutral pH, the DDS exhibited remarkable pH-dependent release, with only 2.5% of Cur released at pH 7.2 and 78% at pH 5.4, demonstrating good selectivity for cancer cells. Moreover, selectivity was also confirmed by cellular uptake studies that showed that the particles can be successfully internalized into HepG2 cells, while no internalization occurs in normal L02 hepatocytes [174]. Other active targeting techniques used FA as the cancer-cell-targeting agent. Danafar’s group reported magnetite NPs coated with BSA and FA for Cur delivery [175]. Although the drug-loading capacity of the proposed DDS was small, it demonstrated good selectivity towards HepG2 cancer cells. Cellular uptake studies showed that the FA-modified DDS could be more efficiently internalized into HepG2 cells compared to their FA-free analogs. Apoptosis assay also indicated a higher rate of cell death in the case of FA-modified DDS. The release of Cur from the DDS was pH dependent, with 70% of Cur released at pH 4.5 after 30 h compared to 55% at pH 7.4 [175]. Another FA-modified DDS comprised of superparamagnetic magnetite NPs and quantum dots was developed. The release of Cur followed a pseudo-second-order kinetics model and was pH-dependent, with approximately twice as much Cur released at pH 5.5 compared to pH 7.4.

Cytotoxicity studies were performed on a breast cancer line and an osteosarcoma cell line and revealed that, at low concentrations, the DDS exhibited better cytotoxicity compared to Cur alone. Moreover, the selectivity of the FA-modified DDS was demonstrated by cytotoxicity tests, because the toxicity of the FA-modified system was greater than that of the unmodified carrier [176].

**Table 2:** Magnetic nanoparticles for the targeted delivery of chemotherapeutic drugs.

Carrier, Drug	Cancer	Targeting	Size (nm)	LC	LE	Release Mechanism	Ref.
Fe_3_O_4_/BNN/PEG, DOX	Liver	Magnetic	151–216	19 ± 0.54%	83 ± 0.33%	pH dependent	[145]
Achiral nanorobot, DOX	Breast, liver	Magnetic, macrophages	40000	-	45%	pH dependent	[151]
Mesoporous Fe_2_O_3_–Au, DOX	Lung	Magnetic	100	-	-	pH dependent	[146]
Fe_3_O_4_/chitosan aerogel, DOX	Osteosarcoma	Magnetic	-	40%	-	pH swelling, AMF	[130]
Fe_3_O_4_/CMCS/ALS, DOX	Breast	Magnetic	90–170	48.68%	86.23%	pH dependent	[147]
Fe_3_O_4_/Nylon/DOX	Lung	Magnetic	28 ± 4	73.2%	-	pH dependent	[153]
Fe_3_O_4_/SiO_2_-AP-DNA, DOX	Breast	Magnetic, enzymatic	70	-	-	Enzymatic	[123]
Fe_3_O_4_/SiO_2_-CS-FA, DOX	Cervical	Magnetic, Active, FA	100	-	15%	Redox, pH dependent AMF	[128]
Fe_3_O_4_/ SiO_2_/DOX	Breast	Magnetic	5–10	62%	-	pH, temperature dependent	[154]
Fe_3_O_4_/L-cys/DOX	Melanoma	Magnetic	3–34	-	-	pH dependent	[155]
Fe_3_O_4_/CaCO_3_/DOX	Breast, cervical	Magnetic	135	1900 µg/mg	-	pH dependent	[156]
CoFe_2_O_4_/MoO_2_/PMMA/DOX/TD, DOX	Liver	Magnetic	20	20%	-	AMF, photothermal	[131]
Fe_3_O_4_ /HT/TA, DOX	Colorectal	Magnetic	70	8.17%	51%	pH dependent	[152]
Fe_3_O_4_/FA, DOX	Colorectal	Magnetic, Active, FA	10	7.15%	95%	Magnetic, pH dependent	[132,157]
Fe_2_O_3_/ChitoPEG, DOX	Colorectal	Magnetic	148.9	-	-	Redox responsive	[129]
Lipo/SPION/ICG/cRGD, DOX	Fibrosarcoma	Magnetic, Active, cRGD	166 ± 42	-	47.5%	AMF	[133]
MNC/PDO, CIS	Cervical, breast	Magnetic	90–100	0.067%	-	pH dependent	[161]
MNP/PEG/IL,CIS	-	Passive	15.1 ± 1.7	36 g Pt/ g Fe	-	Cytosolic media	[158]
Fe_2_O_3_/NGO, CIS	Glioblastoma	Magnetic	10	37%	-	-	[159]
Fe_3_O_4_/ SiO_2_/dicarboxylic acid groups CIS	Pancreatic	Passive	54 ± 9	11%	23%	pH dependent	[160]
Fe_3_O_4_/SiO_2_/EDTA/HA, CIS	Colorectal	Magnetic, Active, HA	70–100	34.11%	82.85	pH dependent	[162]
Fe_3_O_4_/ASA/PEG/TAT, CIS	Nasopharyngeal	Magnetic, Active, TAT	49.42 ± 9.5	-	-	-	[118]
Fe_3_O_4_/FA/CBD, CIS	Nasopharyngeal	Magnetic, Active, FA, CBD	20 ± 1	6.32%	61.35%	-	[163]
Fe_3_O_4_/CS, OXA, IRI	Colon	Passive	36.77	-	-	-	[164]
Fe_3_O_4_/Ma/C, OXA	Colon	Magnetic	34 ± 10	40%	-	pH dependent	[165]
Fe_3_O_4_/Au/Herceptin, OXA	Gastric	Magnetic, Active, Ab	8–20	-	-	pH dependent	[166]
Fe_3_O_4_/PLGA/anti-CD133 Ab, OXA	Colorectal carcinoma	Active, Ab	166 ± 25	22%	44%	-	[119]
Fe_3_O_4_/l-lysine, MTX	Breast	Passive	43.72 ± 4.73	8.9%	-	Enzymatic	[124]
Fe_3_O_4_/Arg, MTX	Breast	Passive	26.99 ± 7.31	8.25 ± 0.29%	-	Enzymatic	[125]
Fe_3_O_4_/Gly, MTX	Breast	Passive	46.82 ± 5.03	4.2%	-	pH dependent, enzymatic	[126]
Fe_3_O_4_/BSA, MTX	Breast	Passive	105.7 ± 3.81	3.5%	-	Enzymatic	[127]
Fe_3_O_4_/ PHBV, MTX	Colorectal	Passive	90	6.79 ± 0.01%	84%	-	[167]
Fe_3_O_4_/Cat/Ciclo, MTX	Osteosarcoma	Passive	20–80	8.92%	89.27%	pH dependent	[168]
Fe_3_O_4_/DPA/PEG/HA, MTX	Lung	Active, HA	103	42.94%	88.11%	pH dependent	[169]
Fe_3_O_4_/FRab, MTX	Cervical	Active, FR ab	50–100	-	90.62%	pH dependent	[170]
Fe_3_O_4_/Cur-LDH/PDO, Cur	Liver	Passive	179.4 ± 29.8	38%	-	pH dependent	[173]
Fe_3_O_4_/C-VB-PEGMA, Cur	Liver	Active, VB	10.3 ± 1.3	25%	67.7%	pH dependent	[174]
Fe_3_O_4_/BSA, Cur	Liver	Active, FA	60.21 ± 12.32	5%	-	pH dependent	[175]
Fe_3_O_4_/GQD, Cur	Breast, osteosarcoma	Active, FA	34.3	-	-	pH dependent	[176]
Fe_3_O_4_/PVA/LDH, SOR Fe_3_O_4_/PEG/LDH, SOR	Liver	Passive	19 17	54% 69%	-	pH dependent	[177]
Fe_3_O_4_/PVA/LDH, SOR	Liver	Passive	19	87%	-	pH dependent	[178]
Fe_3_O_4_/Alg, SOR	Liver	Passive	10–15	-	58.8%	Biphasic release	[179]

67 μg of Pt per 100 mg of MNC@PDO, mg Pt/mg DDS, mg OXA/mg magnetite. BNN—boron nitride nanosheets; CMCS—carboxymethyl chitosan; ALS—aminated lignosulfonate; CS—chitosan; FA—folic acid; AMF—alternating magnetic field; PMMA—poly(methacrylic acid); TD—1-tetradecanol; ChitoPEG—methoxy polyethylene glycol grafted to chitosan; Lipo—lipsome; SPION—superparamagnetic iron oxide nanoparticle; ICG—indocyaninde green; CIS—cisplatin; MNC—magnetic nanoclusters; PDO—polydopamine; IL—ionic liquid; NGO—nanographene oxide; ASA—aldehyde sodium alginate; TAT—transcription activator peptide; CBD—intracellular aggregation ability peptide; OXA—oxaliplatin; IRI—irinotecan; Ab—antibody; PLGA—poly(lactide-co-glycolide); DPA—dopamine; MTX—methotrexate; Gly—glycine; FR—folate receptor; Cat—cationic polymer; cyclo—cyclodextrin; Arg—arginine; BSA—bovine serum albumin; Cur—curcumin; LDH—layered double hydroxide; VB—4-vinylphenylboronic acid; PEGMA—polyethylene glycol methyl ether methacrylate; GQD—graphene quantum dots; SOR—sorafenib; PVA—polyvinyl alcohol; LDH—layered double hydroxide; Alg—alginate; L-cys—cysteine.

### 5.5. Sorafenib

Sorafenib (SOR) presents systemic side effects, low bioavailability, and water solubility [144], which can be limited by encapsulating it in DDSs.

Several attempts have been made to use magnetite NPs for the delivery of SOR in the treatment of hepatocellular carcinoma. Anionic compounds, such as LDH or alginate, were used to functionalize the core particles and allow SOR binding. Pastorin’s group developed superparamagnetic iron oxide NPs modified with polyvinyl alcohol (PVA) or PEG and double-layered magnesium-aluminum hydroxide for the delivery of SOR [177]. PEG-coated NPs demonstrated a higher drug-loading capacity, smaller hydrodynamic diameter, and narrower size distribution. Both carriers exhibited a sustained, pseudo-second-order kinetics, pH-dependent release behavior, in vitro. Due to PEG’s higher solubility in water compared to PVA, the release from the PEG-modified particles was faster than from the PVA-modified ones. The same group reported magnetite NPs modified with PVA and zinc-aluminum LDH [178]. Compared to the previous approach, the drug-loading capacity was significantly increased. The release of SOR from the SOR-functionalized carriers was higher than from a simple physical mixture of SOR and carriers demonstrating the advantage of the nanoformulation. The release was pH-dependent and followed pseudo-second-order kinetics [178]. Cytotoxicity studies were carried out on fibroblasts and HepG2 hepatocellular carcinoma cells and demonstrated that all nanoformulations had superior cytotoxicity compared to SOR alone on cancer cells, while displaying good biocompatibility with fibroblasts [177,178]. Another approach consisted of the encapsulation of SPIONs into alginate microspheres containing SOR. The system demonstrated acceptable drug-loading efficiency and a biphasic release profile, with 54% of the total SOR released in the first hour. Since the release profile was studied at pH 7.4, which is the normal pH of serum, not of the tumor environment, this could raise the question of the safety of the system, since SOR could be released in serum before reaching the tumor. The formulation exhibited cytotoxicity towards HepG2 cells; however, no comparison to SOR alone was performed [179].

## 6. RNA Delivery Using Magnetic Nanoparticles

Non-coding RNAs, such as miRNA and siRNAs, have recently emerged as promising therapeutic tools for cancer management. The main limitations of RNA use in therapy are related to its limited ability to pass through cellular membranes, due to its hydrophilic nature, negative charge, and instability in the presence of serum enzymes [180]. For these reasons, numerous attempts have been made at encapsulating RNA in DDSs that could enhance its pharmacokinetics and stability.

**Figure 5:**
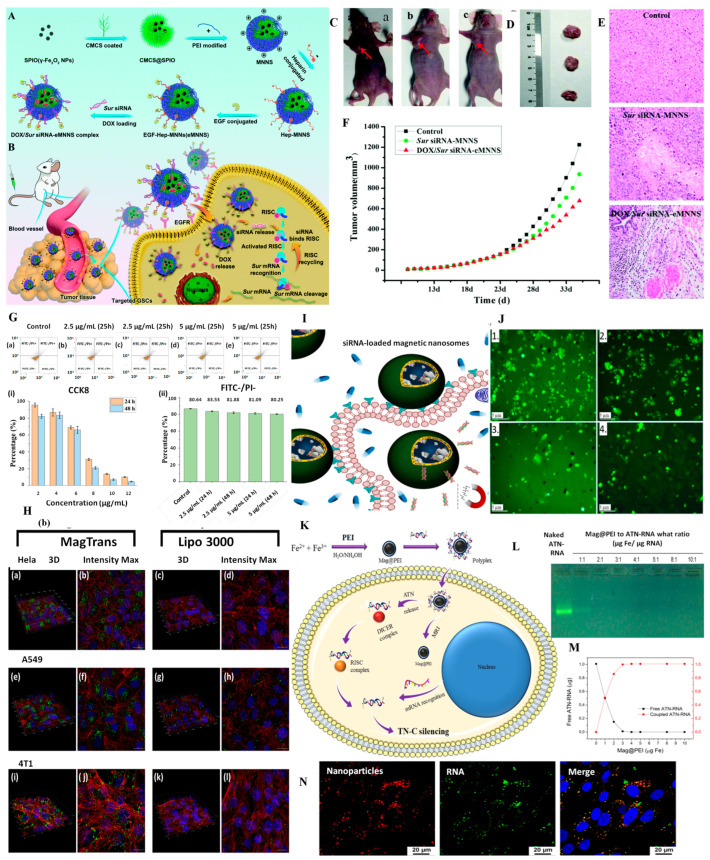
(**A**) Schematic illustration of the fabrication of the EGFR-targeted theragnostic nanoplatform, and (**B**) targeted co-delivery of therapeutic siRNAs and DOX for GSC treatments. (**C**) Typical photographs showing the size of tumors (indicated by arrows) in mice on day 2 weeks with three different treatments. Photo-images of excised tumors from mice bearing glioblastoma U251-induced tumors 2 weeks after different treatments. a: Control; b: Sur siRNA MNNS; c: DOX/Sur siRNA-eMNNS. (**D**) Tumor growth inhibition for the mice bearing glioblastoma U251-induced tumors after treatment with various formulations (*n* = 3). (**E**) Representative histology (H&E) images of the tumor tissue in the control, Sur siRNA-MNNS, and DOX/Sur siRNA-eMNNS groups. (**F**) Active targeting co-delivery of therapeutic Sur siRNA and an antineoplastic drug via epidermal growth factor receptor-mediated magnetic nanoparticles for synergistic programmed cell death in glioblastoma stem cells. Adapted with permission from Ref. [187] Copyright 2023 Royal Society of Chemistry. (**G**) Fluorinated PEG-PEI Coated Magnetic Nanoparticles for siRNA Delivery and CXCR4 Knockdown (a–e) Scatter diagram of HeLa cells apoptosis stained by Annexin V-FITC/PI measured by flow cytometry. (i) Relative cell viability measured by CCK8 kit. (ii) Analysis of cell viability measured by flow cytometry (a–e). (**H**) Results of FAM-siRNA transfection captured by confocal microscopy. Blue is Hoechst33342-stained nuclei, Green is FAM-labeled siRNA NC, and Red is Rhodamine-Phalloidin-stained F-actin (a–d) HeLa, (e–h) A549, (i–l) 4 T1.) Reprinted from Ref. [182] (Open access). In vitro Application of Magnetic Hybrid Niosomes: (**I**) Targeted siRNA-Delivery for Enhanced Breast Cancer Therapy—schematic representation of the experimental protocol. (**J**) Representative fluorescent images (using the Calbryte-520 Assay Kit) showing occurring apoptosis (light green) of BT-474 cells after administration of (1) siRNA/FexOy/NIO with erlotinib, (2) free siRNA with erlotinib, (3) erlotinib and (4) control cells and subsequent magnetic treatment. Reprinted from Ref. [183] (Open access). Nano-mediated delivery of double-stranded RNA for gene therapy of glioblastoma multiforme. (**K**) Schematic diagram of preparation of Mag@PEI /ATN-RNA complexes and its application in RNAi therapy of GBM cells. Binding of ATN-RNA to Mag@PEI NPs. (**L**) Agarose gel electrophoresis of Mag@PEI/ATN-RNA complexes at the different mass ratio. (**M**) Binding capability of Mag@PEI NPs towards ATN-RNA recorded using Nanodrop. (**N**) Colocalization of Mag@PEI NPs and ATN-RNA in U-118 cells. The representatives of the colors are blue (Hoechst 33342) for nuclei, green (FITC) for ATN-RNA, and red (ATTO550) for Mag@PEI nanoparticles. Reprinted from Ref. [184] (Open access).

In the development of magnetic DDSs for RNA delivery, magnetite has been the most commonly used magnetic core. Cationic polymers, especially polyethyleneimine (PEI), were generally used for the functionalization of the cores, due to their ability to interact electrostatically with the negatively charged RNA cargo. MicroRNAs and siRNAs were loaded into the magnetic carriers. Some studies also included chemotherapy drugs, such as DOX (Figure 5A–F) together with siRNA for enhanced in vivo efficiency. The release of the RNA was mostly achieved by alternating magnetic field (AMF) triggering, but also by pH-dependent mechanisms. Targeting was generally passive, with some examples of magnetic or active targeting also present.

Controlled delivery of miR-1484 mimic was achieved by magnetite NPs covered with a thermo-liable Diels-Alder cycloadduct that released the RNA in A549 lung cancer cells in the presence of an AMF that led to heating and subsequent cleavage of the Diels-Alder bond. The cytotoxicity of the DDS was higher towards A549 in the presence of AMF than in its absence, indicating its importance in the release of the RNA cargo. A high concentration of NPs was used to compensate for the relatively low RNA loading efficiency, which could pose safety concerns [134]. Two similar approaches for the delivery of miR-34 for the treatment of neuroblastoma were developed using PEI [135] and PEI and tripolyphosphate (TPP) [136], respectively. The carriers were similar in size and demonstrated AMF-triggered release of the RNA due to an increase in temperature and shell softening. TPP conjugation decreased the inherent toxicity associated with PEI. Genetic analyses demonstrated that both approaches led to the inhibition of the MYCN oncogene.

Magnetic guidance was used in several examples for the delivery of siRNA to tumor cells. Iron oxide NPs were functionalized with caffeic acid, calcium phosphate, PEG, and siRNA and their cytotoxic potential was evaluated in an HER2-positive breast cancer cell line. HER2 expression levels were decreased after incubation with the DDS and expression levels decreased further when a magnetic field was applied. Cell internalization was also increased in the presence of a magnetic field [181]. In order to reduce the toxicity of PEI, while also maintaining the DDS’ (FFP@MNP) capacity to escape endosomal entrapment, Zhang et al., developed magnetite NPs functionalized with PEI and fluorinated PEG. Figure 5G shows the effect of FPP@MNPs on cell viability, which was characterized by the CCK8 kit. Different concentrations of FPP@MNP were added to HeLa cells and then cultured for 24 h or 48 h. It was observed that the viability of HeLa decreased with the incubation time and FPP@MNPs were toxic for concentrations ≥ 8 μg/mL. The results of FPP@MNPs-induced HeLa cell apoptosis are shown in Figure 5G(a–e), and the analysis of cell viability is shown in Figure 5G(ii). The cells were scanned layer by layer and three-dimensionally reconstructed by laser confocal microscopy (Figure 5H). HeLa, A549, and 4 T1 cells were transfected with FPP@MNPs under external magnetic field enhancement (MagTrans). The commercial nucleic acid vector Invitrogen Lipofectamine 3000 (Lipo3000) was used as the control. For each group of cells, it is evident that the amount of siRNA inside MagTrans-treated cells is significantly higher than Lipo3000 transfected cells. For Lipo3000-transfected cells, the distribution of siRNA can be observed in almost every cell, but the amount of siRNA contained in a single cell is far less than that of cells treated by MagTrans. Transfection methods used were magnetic field-enhanced F7-PEG-PEI@MNP transfection (MagTrans) or Lipo3000 transfection (Lipo 3000) [182]. Iron oxide NPs were integrated into non-ionic surfactant vesicles (niosomes) and used for the combined delivery of siRNA and transtuzumab/erlotinib to breast cancer cells. The schematic representation of the experimental protocol for DDS fabrication is represented in Figure 5I. High siRNA encapsulation was obtained, and in the case of both drugs, siRNA significantly increased the activation of apoptotic pathways, especially after the application of a magnetic field. The representative fluorescent images showing the occurrence of apoptosis of BT-474 cells after administration of siRNA/FexOy/NIO with erlotinib, free siRNA with erlotinib, erlotinib, and control cells are presented in Figure 5J [183].

PEI was also used as a shell for magnetite NPs used in the delivery of siRNA. Zhang et al., reported a DDS for the combined delivery of two siRNAs for applications in oral cancer treatment. The NPs demonstrated a high affinity for RNA and gene silencing potential comparable to that of lipofectamine, which was used as a positive control. Moreover, cancer cell migration was also impaired by the DDS [137]. Another approach using PEI as a modifier for the delivery of siRNA in the treatment of glioblastoma was also reported. Magnetic nanoparticles modified with PEI and ATN-RNA were synthesized, and the schematic diagram of preparation and its application in RNAi therapy of glioblastoma cells is presented in Figure 5K. The optimized complexes of Mag@PEI/ATN-RNA were submitted to an agarose gel electrophoresis assay to visualize the linking of ATN-RNA to Mag@PEI NPs (Figure 5L). Analysis revealed that two weight equivalents were sufficient to bind almost all of the ATN-RNA used in the experiment. However, to gain a deeper insight into this process, ATN-RNA concentration in the supernatant was investigated by UV-Vis spectroscopy (Figure 5M). A colocalization analysis was also performed to further demonstrate the transfer of Mag@PEI/ATN-RNA complexes into glioblastoma cells, demonstrated via a colocalization analysis (Figure 5N). A visible colocalization between the ATTO 550-labeled Mag@PEI nanoparticles (red color) and the FITC-labeled ATN-RNA (green color) in the cytoplasm was obtained, suggesting high transfection efficiency of the synthesized nanoparticles [184]. Another study reported gelatin-covered magnetite NPs loaded with siRNA, which demonstrated cytotoxicity towards Caco-2 colorectal cancer cells [185]. Their toxicity was higher or similar to that of HiPerFect^®^, a commercial RNA transfection agent. However, no gene expression or cell migration assays were reported.

Active targeting of siRNA and chemotherapeutic drugs such as paclitaxel and DOX was achieved by coupling functionalized magnetic NPs with folate and T7 peptide [186] and epidermal growth factor receptor (EGFR) ligand, respectively [187]. The complex approach to paclitaxel and siRNA delivery involved the combination of magnetic NPs with polylactic acid-PEG and FA, peptide T7, and chitosan-spermine. The DDS exhibited higher encapsulation efficiency for siRNA than for the drug, and the release behavior was pH-dependent. Transfection efficiency was similar to that of lipofectamine in MCF-7 breast cancer cells. Active delivery of survivin siRNA and DOX was achieved by using a DDS comprised of maghemite NPs functionalized with carboxymethyl chitosan (CMCS), PEI, heparin, and EGF, labeled as eMNNS. The preparation of eMNNS was performed in three main steps: (1) Synthesis of the core followed by TPP-mediated encapsulation of CMCS; (2) PEI coating via EDC/NHS crosslinking; and (3) conjugation of Heparin and EGF with MNNS to form eMMNS (Figure 5A), while the targeted co-delivery of therapeutic siRNAs and DOX for GSC treatments is presented in Figure 5B. In vitro analyses were conducted to evaluate the tumor-suppressing effect of the DDS to inhibit the growth of glioblastoma stem cells (GSCs). In vivo experiments were conducted on BALB/c nude mice bearing glioblastoma U251 cell-induced tumors (Figure 5C,D). The mice were divided into three groups and administered different formulations to assess the safety and efficacy of eMNNS for cancer therapy. The first group was treated with Sur siRNA-MNNS (Figure 5C(a)), the second group with DOX/Sur siRNA-eMNNS (Figure 5C(b)), and the third group served as a control group (Figure 5C(c)). The results showed that the tumor size of the mice treated with Sur siRNA-MNNS decreased compared to the control group, while the mice treated with DOX/Sur siRNA-eMNNS exhibited the smallest tumor size and the best anti-tumor capacity. Moreover, histological analysis revealed extensive necrosis areas within the tumor mass treated with DOX/Sur siRNA-eMNNS. It was observed that the tumor size of the mice treated with Sur siRNA-MNNS decreased compared to the control group, and the mice treated with DOX/Sur siRNA-eMNNS exhibited the smallest tumor size and the best anti-tumor capacity (Figure 5C–E). Moreover, the histological analyses revealed extensive necrosis areas within the tumor mass treated with DOX/Sur siRNA-eMNNS (Figure 5F) [187].

Some examples of magnetic nanoparticles applied for RNA delivery are presented in Table 3.

## 7. Theragnostic Agents

Theragnostic approaches are emerging as innovative techniques in cancer management. Nanotechnology offers a vast array of nanosized devices known as nanotheragnostics, which combine diagnostic and therapeutic capabilities for real-time disease monitoring and treatment at the cellular and molecular levels. To effectively diagnose and treat diseases using nanotheragnostics, it is crucial for these nanomedicines to circulate throughout the body without being eliminated by the immune system.

One important aspect of nanotheranostics is their ability to target specific organs or tissues by attaching biological ligands to their surface. This targeting mechanism improves the efficiency and specificity of drug delivery, ensuring that the therapeutic agents reach the intended site of action [188]. Combining drug delivery and imaging can achieve early detection and enhanced treatment [189].

In the field of diagnostics, non-invasive techniques have gained significant attention. MNPs are being used as novel diagnostic agents, particularly in MRI for imaging various diseases, providing detailed anatomical and functional information. However, recent advancements have enabled the utilization of MNPs not only for imaging but also for drug targeting to the cellular membrane and for multi-modal imaging. By incorporating therapeutic agents and imaging capabilities into a single nanotheragnostic system, it becomes possible to simultaneously monitor the disease progression and deliver treatment directly to the affected cells or tissues. This approach holds promise for more effective and personalized medicine, as it allows for real-time monitoring of treatment response and adjustment of therapeutic strategies as needed in personalized medicine [190].

In recent years, MNPs and nanocomposites have been employed for the development of theragnostic agents. In Table 4, some examples of theragnostic approaches using MNPs are summarized.

## 8. Perspectives and Conclusions

Chemotherapy is currently one of the primary methods for treating cancer, but its effectiveness can be limited by inefficient drug delivery and damage to normal tissues caused by uncontrolled drug release. The use of RNA-mediated cancer therapy is a novel therapeutic strategy that still faces some limitations. Nanomedicine delivery systems have been developed to address these challenges by improving the specificity and efficiency of drug delivery to cancer cells while minimizing harm to healthy tissues. Magnetic nanoparticles have been extensively studied in recent years for their potential applications as DDSs, diagnosis, or theragnostic tools. Different types of magnetic nanomaterials can be used, such as magnetic pure metals, magnetic oxides, multicomponent MNPs (MCs), or magnetic nanocomposites, each exhibiting unique properties. To be used in medical applications, these NPs need to meet minimal requirements such as biocompatibility, improved stability in biological fluids, adequate size, and superparamagnetism.

Moreover, adequate drug loading and controlled release need to be achieved for the successful use of magnetic DDSs in drug delivery applications. Uncontrolled drug release can lead to toxicity and damage to healthy tissues. Thus, researchers are exploring various strategies for achieving controlled drug release, such as pH-responsive or stimuli-responsive DDS. DDSs have been developed for the targeted delivery of chemotherapeutics such as doxorubicin, platinum compounds, methotrexate, sorafenib, and curcumin, as well as for RNA-mediated therapeutics. Their effects were mostly tested in vitro, with a few examples of strategies also tested in vivo. The magnetic properties of MNPs represent an advantage due to the possibility of applying a magnetic field to guide them. Moreover, the application of magnetic fields can also improve the anti-cancer efficiency, by using hyperthermia or magneto-mechanical actuation of MNPs. Magnetic hyperthermia is a well-known technique that can be combined with drug delivery to enhance tumor-killing properties by increasing temperature at the tumor site. Apart from this, magneto-mechanical actuation of MNPs has recently emerged as a tool in cancer treatment. In this technique, an alternating magnetic field is applied to MNPs, causing them to vibrate and produce mechanical alterations the cells. In the future, this technique could be combined with drug delivery to enhance cancer cell destruction.

Theragnostic approaches have also been developed for combined drug delivery and imaging. This technique offers perspectives for personalized medicine using MNPs. Simultaneous imaging and treatment could potentially lead to better delivery of the drug in the proximity of the tumor, real-life treatment monitoring, and improved outcomes for the patient.

There are several ways to incorporate MNPs into clinical practice. By leveraging nanotechnology, interdisciplinary collaboration, and standardization, MNPs can offer early disease detection, targeted delivery of treatments to diseased areas, and non-invasive monitoring of novel therapies. The review briefly discusses the importance of MNPs (including SPIONs) and their synthesis, stability through surface modification, and applications for DDSs and medical treatment. The size, shape, and surface chemistry of MNPs impact their pharmacokinetics and toxicity. By fine-tuning these properties, improved passive, active, and magnetic targeting approaches can be achieved. Attention must be given to the choice of targeting agent and the attachment method for MNPs to maximize sensitivity in active targeting approaches.

In the future, more research needs to be carried out in the field of MNPs to fully assess their toxicity, drug-loading potential, and in vivo properties. However, the existing results show the promising impact of MNP-based DDSs for the treatment of cancer. Polymer-functionalized MNPs show perspective for future studies due to their increased biocompatibility, drug-loading capacity, and improved stability. The preliminary results obtained in this field show promise for improved drug delivery and personalized medicine; however, many challenges still remain until the advent of the use of magnetic DDSs in a clinical setting. With recent advances in the synthesis and modification of MNPs, as well as increased awareness of the potential immunomodulatory effects of nanoscale heat, MNPs continue to hold promise as a valuable tool for both physicians and patients.

## Figures and Tables

**Table 1:** Drugs commonly prescribed in the treatment of cancers [141,142,143,144].

Drug	Indications	Class	Mechanism of Action	Specific Side Effects
DOX	Acute lymphoblastic leukemia Hodgkin’s lymphoma Breast cancer Ovarian cancer Bladder cancer Bone tumors	Anthracyclines	Intercalation into DNA double-helix Topoisomerase II inhibition Formation of oxygen reactive species	Cardiotoxicity
CIS, OXA	Ovarian cancer Breast cancer Colorectal carcinoma	Alkylating agents	Intercalation into DNA double-helix	Neurotoxicity Nephrotoxicity Ototoxicity
MTX	Non-Hodgkin’s lymphoma Breast cancer Bladder cancer Osteosarcoma	Antimetabolite	Inhibition of folic acid metabolism	Immunosuppression Hepatotoxicity Respiratory failure
SOR	Hepatocellular carcinoma Renal cell carcinoma Thyroid cancer	Multi kinase inhibitor	Inhibition of cell signaling pathways Inhibition of angiogenesis	Rash Arterial hypertension

**Table 3:** Magnetic nanoparticles for the targeted delivery of RNA.

Carrier, RNA	Cancer	Targeting	Size (nm)	LC	EE	Release Mechanism	Ref.
Fe_3_O_4_/DAC, miR-1484 mimic	Lung	Passive	10.1 ± 0.5	2.5 × 10^−10^ moles/mg NPs	8.4%	AMF	[134]
Fe_3_O_4_/PEI/TPP, miR-34a	Neuroblastoma	Passive	20	-	-	AMF	[136]
Fe_3_O_4_/PEI, miR-34a	Neuroblastoma	Passive	10–20	-	-	AMF	[135]
Fe_3_O_4_/PEI, siRNA	Glioblastoma	Passive	8–12	-	90%		[184]
Fe_3_O_4_/PEI, siRNA	Oral	Passive	7.95	-	100%	AMF	[137]
Fe_3_O_4_/Gel, siRNA	Colorectal	Passive	60	-	41.5%		[185]
Fe_2_O_3_-Fe_3_O_4_/Caf/CaP/PEG-Pa siRNA	Breast	Magnetic	14	1.5 ± 0.1 μM			[181]
Fe_3_O_4_/FPP/PEI, siRNA	Cervical	Magnetic	12	-	-		[182]
FexOy/noisome siRNA + erlotinib siRNA + transtuzumab	Breast	Magnetic	100	-	99%	-	[183]
Fe_3_O_4_/PCS/PPF/PPT siRNA/PTX	Breast	Active, FA, T7 peptide	197 ± 16	-	68.52% (siRNA) 41.31 ± 3.6% (PTX)	pH dependent	[186]
Fe_3_O_4_/CMCS/PEI/Hep siRNA/DOX	Glioblastoma	Active, EGF	40–50	3.86% (DOX)	-	-	[187]

Gel—gelatin; DAC—Diels-Alder cycloadduct; PEI—polyethyleneimine; TPP—tripolyphosphate; PCS—Poly-lactic acid-Chitosan-Spermine; PPF—poly-lactic acid polyethylene glycol-folate; PPT—Poly-lactic acid-PEG-T7 peptide; CMCS—carboxymethylchitosan; Hep –heparin; EGF—epidermal growth factor; Caf—caffeic acid; CaP—calcium phosphate; PEG-Pa—polyethylene glycol anionic polymer; FPP—Heptafluorobutyryl-polyethylene glycol-polyethyleneimine.

**Table 4:** Theragnostic agents for the simultaneous treatment and diagnosis of cancer.

Agent	Cancer	Type of action	Ref.
Fe_3_O_4_/Chi/GO	Breast	DOX delivery + MRI	[191]
Fe_3_O_4_/PEG-b-PLA	Breast	DOX delivery + MPI	[192]
SPION/Lipo/Pept	Breast	PTX delivery + MRI	[193]
SPION/Lipo	CNS lymphoma	RTX delivery + MRI	[194]
Zn-doped SPION/Den/Apt/Fluo	Breast	DOX delivery + gene silencing + hyperthermia + NIR/MR imaging	[195]

Chi—chitosan; MRI—magnetic resonance imaging; PEG-b-PLA—polyethylene glycol-block-poly lactic acid copolymer; MPI—magnetic particle imaging; Lipo—liposome; Pept—pH responsive peptide; PTX—paclitaxel; CNS—central nervous system; RTX—rituximab; Den—dendrimer; Apt—aptamer; Fluo—fluorescent dye; NIR—near infra-red.

## Data Availability

Not applicable.
